# Perioperative Drug Treatment in Pancreatic Surgery—A Systematic Review and Meta-Analysis

**DOI:** 10.3390/jcm12051750

**Published:** 2023-02-22

**Authors:** Ingmar F. Rompen, Daniela C. Merz, Karam T. Alhalabi, Rosa Klotz, Eva Kalkum, Thomas M. Pausch, Hendrik Strothmann, Pascal Probst

**Affiliations:** 1 Department of General, Visceral and Transplantation Surgery, Heidelberg University Hospital, 69120 Heidelberg, Germany; 2 Study Center of the German Society of Surgery, 69120 Heidelberg, Germany; 3 Department of Surgery, Cantonal Hospital Thurgau, 8596 Münsterlingen, Switzerland; 4 Department of Surgery, Cantonal Hospital Thurgau, 8501 Frauenfeld, Switzerland

**Keywords:** pancreas, surgery, somatostatin, glucocorticoid, proton pump inhibitors, pancreatic enzyme replacement, insulin

## Abstract

Introduction: Pancreatic resections for malignant or benign diseases are associated with major morbidity and changes in physiology. To reduce perioperative complications and enhance recovery, many types of perioperative medical management have been introduced. The aim of this study was to provide an evidence-based overview on the best perioperative drug treatment. Methods: The electronic bibliographic databases Medline, Embase, CENTRAL, and Web of Science were systematically searched for randomized controlled trials (RCT) evaluating perioperative drug treatments in pancreatic surgery. The investigated drugs were somatostatin analogues, steroids, pancreatic enzyme replacement therapy (PERT), prokinetic therapy, antidiabetic drugs, and proton pump inhibitors (PPI). Targeted outcomes in each drug category were meta-analyzed. Results: A total of 49 RCT were included. The analysis of somatostatin analogues showed a significantly lower incidence of postoperative pancreatic fistula (POPF) in the somatostatin group compared to the control group (OR 0.58, 95% CI: 0.45 to 0.74). The comparison of glucocorticoids versus placebo showed significantly less POPF in the glucocorticoid group (OR 0.22, 95% CI: 0.07 to 0.77). There was no significant difference in DGE when erythromycin was compared to placebo (OR 0.33, 95% CI: 0.08 to 1.30). The other investigated drug regimens could only be analyzed qualitatively. Conclusion: This systematic review provides a comprehensive overview on perioperative drug treatment in pancreatic surgery. Some often-prescribed perioperative drug treatments lack high quality evidence and further research is needed.

## 1. Introduction

Pancreatic resections, mostly performed due to pancreatic cancer treatment or chronic pancreatitis by pancreatoduodenectomies (PD), distal pancreatectomies (DP), or total pancreatectomies (TP), are associated with a morbidity of 50.4% [1,2]. Most common and worrisome complications after pancreatic resections contain postoperative pancreatic fistula (POPF), delayed gastric emptying (DGE), post pancreatectomy hemorrhage (PPH), and bile leak [3].

Somatostatin analogues are used to lower the amount of pancreatic juices’ secretion and therefore may have an influence on POPF [4]. Glucocorticoids do reduce the inflammatory and stress response, a possible mediator of post-pancreatectomy complications [5]. Erythromycin may has an effect on pyloric relaxation and is used for treatment of DGE [6]. Inhibition of gastric acid production by proton pump inhibitors (PPI) may decrease the rate of anastomotic bleeding [7]. Furthermore, PD, DP, or TP are associated with endocrine and exocrine insufficiency. Oral replacement of lipase and pancreatic amylase is used to improve dietary functions, whereas insulin therapy can be given to treat endocrine insufficiency [8]. PPI may restrain the pancreatic remnant to shrink and therefore may have a positive effect in obtaining exo- and endocrine function [9].

So far, there is no systematic review and meta-analysis on most of these medications. With this systematic review with meta-analysis, the available evidence for the above-mentioned medications on the perioperative outcome in patients undergoing pancreatic surgery is provided.

## 2. Materials and Methods

This systematic review and meta-analysis was conducted according to a prior published study protocol (PROSPERO 2021, CRD42021232211) and according to the Preferred Reporting Items for Systematic Reviews and Meta-Analysis (PRISMA) checklist [10]. There was no external source of funding. According to the recommendations of the Cochrane Collaboration, the search, study selection, and data extraction were performed by two independent reviewers [11]. Disagreement was solved by consensus with a third reviewer.

### 2.1. Literature Search

The electronic bibliographic databases Medline, Embase, Cochrane Central Register of Controlled Trials (CENTRAL), and Web of Science were systematically searched throughout November 2022. For Medline, CENTRAL, and Web of Science, a comprehensive search strategy was used as described elsewhere [2]. For Embase, a specific search strategy was used which is displayed in the Supplementary Materials. No language restrictions were applied.

### 2.2. Study Selection and Data Extraction

All randomized controlled trials (RCT) evaluating perioperative (pre-, intra- and postoperative) drug treatments in pancreatic surgery were included. All trials investigating adult patients undergoing any kind of pancreatectomy that include a risk for relative exocrine or endocrine insufficiencies, i.e., PD, DP, or TP, were considered for inclusion. Studies with a minority of other interventions including enucleations, papillectomies, central pancreatectomies, or draining procedures such as cysto-jejunostomy were included as well and listed in the baseline tables.

The investigated drugs were somatostatin analogues, steroids, pancreatic enzymes replacement therapy (PERT), prokinetic therapy, antidiabetic drugs, and PPI. Any comparisons to the above-mentioned drugs were considered including placebo.

For each drug analyzed, mortality, POPF, bile leak, PPH, DGE, intraabdominal abscess/fluid collection, length of hospital stay (LOS), and operation time were investigated. Additional outcomes for pancreatic enzymes included change in body weight, change in coefficient of fat absorption (CFA), stool fat excretion, stool volume and frequency, bowel habits, symptoms of maldigestion, measurements of intestinal absorption, and nutritional status. Nasogastric tube removal days and the gastric motility index were assessed for prokinetic drugs. Blood glucose level, total amount of insulin required for glycemic control, and postoperative mortality were analyzed for the antidiabetic drug therapy. Last, additional outcomes for PPI included gastrin assay, pancreas volumetry, nutritional status, pancreatic endocrine and exocrine function (serum insulin levels, stool, elastase level), and postoperative volume change in pancreatic remnant. Besides the above-mentioned outcomes, a set of baseline data were extracted: First author, journal, year of publication, region of publication, type of operation, type of disease, age, and gender of patients. Definitions of primary outcomes, DGE, and POPF were extracted separately and are provided in the Supplementary Materials.

### 2.3. Critical Appraisal

Risk of bias was assessed independently by two reviewers using the Cochrane Collaboration tool for assessing risk of bias 2.0 [12]. The tool includes five standard domains of bias: randomization process, deviations from intended interventions, missing outcome data, measurement of the outcome, and selection of the reported result. Any disagreement was resolved by consultation with a third reviewer.

Furthermore, for each pooled outcome the certainty of evidence was rated to be very low, low, moderate, or high by using the “Grading of recommendations, assessment, Development and Evaluation” System (GRADE) [13].

### 2.4. Statistical Analysis

Trials investigating the same drug were pooled. A sub-analysis for studies using the official ISGPS criteria was performed where applicable [14]. Furthermore, a sensitivity analysis was performed for DP versus PD where applicable. Odds ratios (OR) and 95%-CI were calculated for dichotomous outcomes using the Mantel–Haenszel (M-H) method. Continuous outcomes were pooled as mean difference and 95%-CI with the inverse variance method. If trials only reported medians or ranges, the methods described by Hozo, 2005 were applied to calculate means and standard deviations (SD) from the values reported [15]. Meta-analyses were performed with program R (Version 4.2.0). Forest plots were used for the graphical presentation of effect estimates. Publication bias was explored by funnel plotting if more than 10 trials were pooled. For all outcomes, a random-effect model was applied to account for methodological and clinical differences. Statistical heterogeneity among the effect estimates of the included trials was evaluated using the I^2^ statistic. I^2^ less than 25% were considered to indicate low heterogeneity and an I^2^ > 75% to indicate high heterogeneity [16].

## 3. Results

### 3.1. Literature Search

**Figure 1:**
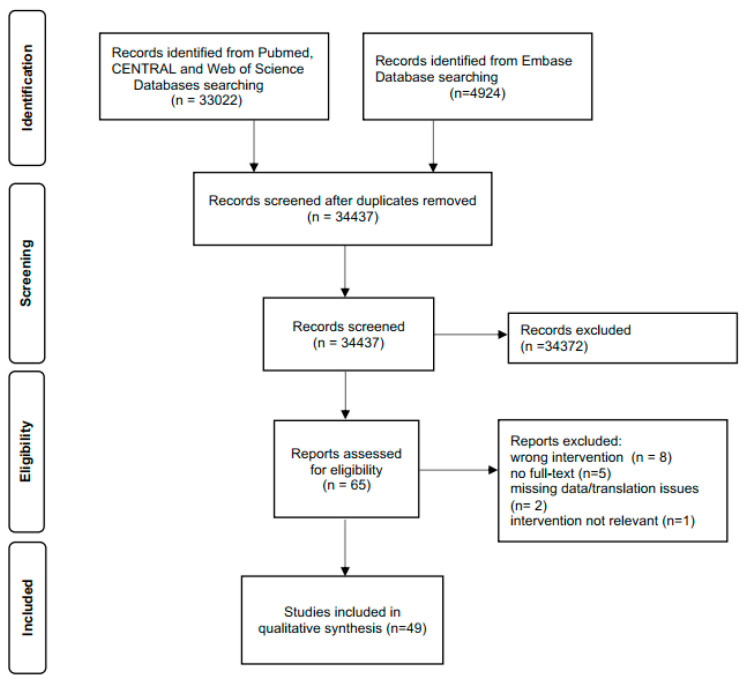
PRISMA Flowchart.

A total of 34,437 articles were evaluated. A detailed description of the screening process can be seen in the PRISMA Flowchart (Figure 1). Finally, 49 trials fulfilled the inclusion criteria and were therefore included in the final qualitative and quantitative analysis.

### 3.2. Somatostatin Analogues

A total of 33 randomized controlled trials including 3742 patients reported on the perioperative outcomes of somatostatin use versus placebo after pancreatic resections (Table 1) [17,18,19,20,21,22,23,24,25,26,27,28,29,30,31,32,33,34,35,36,37,38,39,40,41,42,43,44,45]. Both DP as well as PD were studied. A minority of patients after total pancreatectomy were also included in this analysis. There was no difference in baseline characteristics. Risk of bias assessment resulted in low risk of bias in 3 studies, some concerns in 8 studies, and a high risk in 22 studies. Details can be seen in the Supplementary Materials.

**Figure 2:**
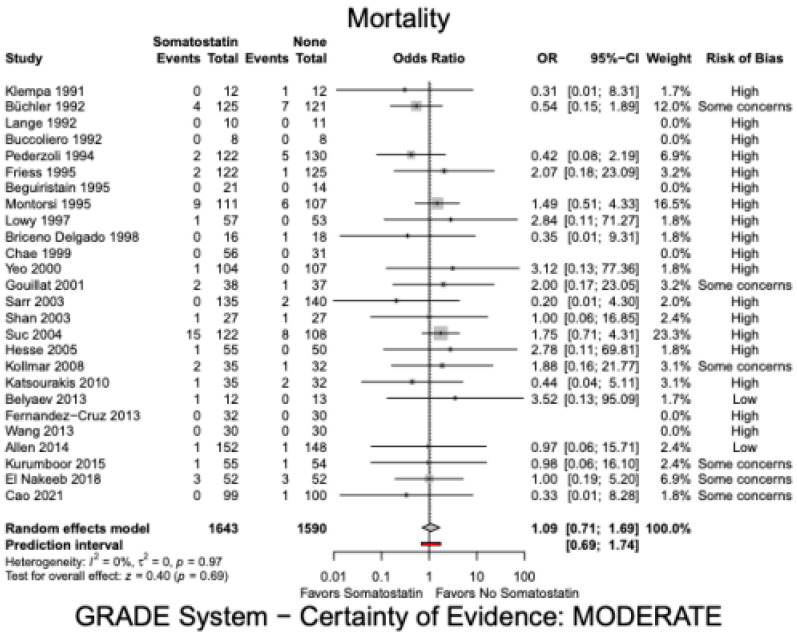
Mortality Somatostatin analogues.

**Figure 3:**
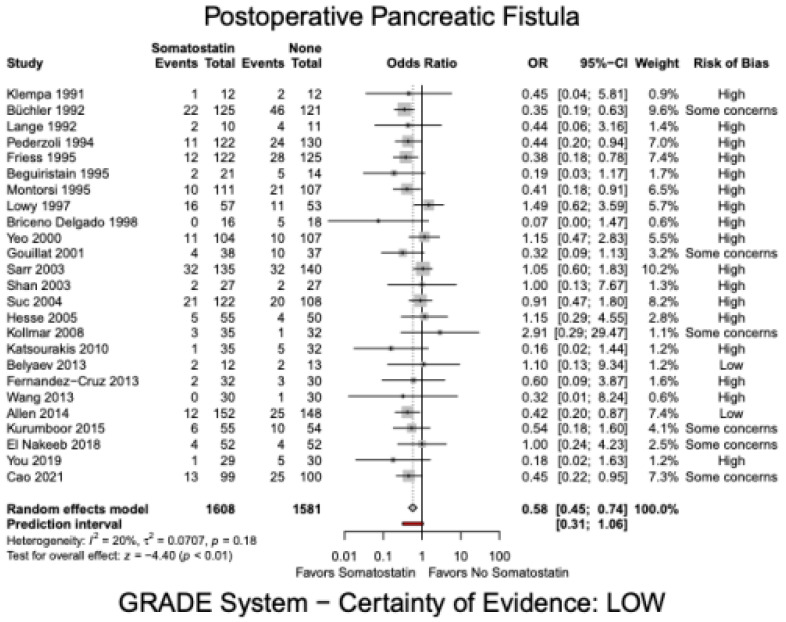
Postoperative Pancreatic Fistula, Somatostatin analogues.

There was no significant difference in mortality in the somatostatin group versus the control group (OR 1.09, 95% CI 0.71 to 1.69, I^2^ = 0%, Figure 2). The certainty of evidence was moderate. Incidence of POPF was significantly lower in the somatostatin group compared to the control group (OR 0.58, 95% CI: 0.45 to 0.74, I^2^ = 20%, Figure 3). However, the certainty of evidence was low. This effect was consistent with a sub-analysis of POPF (Grade B and C) in studies that used the ISGPS criteria (OR 0.48, 95% CI: 0.31–0.73, I^2^ = 0%) [17,23,25,27,34,45]. The sub-analysis included six studies with a total of 833 patients and the certainty of evidence was low. Sensitivity analysis showed a lower rate of POPF in the somatostatin analogue group compared to the control group for both, PD (1842 patients, OR 0.67, 95% CI: 0.52–0.86, I^2^ = 1%, Supplementary Materials) and DP (199 patients, OR 0.35, 95% CI:0.14 to 0.86, I^2^ = 3%, Supplementary Materials). Overall complications failed to show a significant difference (OR 0.76, 95% CI: 0.56 to 1.02, I^2^ = 49%). The certainty of evidence was again low.

There was no significant difference in bile leak (OR 0.85, 95% CI: 0.47 to 1.55, I^2^ = 0%), PPH (OR 1.02, 95% CI: 0.70 to 1.49, I^2^ = 0%), DGE (OR 1.03, 95% CI: 0.73 to 1.45, I^2^ = 0%), intraabdominal fluid collection or abscess formation (OR 1.00, 95% CI: 0.72 to 1.40, I^2^ = 0%), and LOS (MD-2.14 days, 95% CI: −4.40 to 0.12, I^2^ = 99%) with a low to very low certainty of evidence according to the GRADE evaluation.

### 3.3. Glucocorticoids

Two trials including a total of 93 patients were included in this analysis comparing glucocorticoids versus placebo. The trial of Laainen et al. analyzed high-risk patients after PD, whereas the trial of Antila et al. analyzed patients after distal pancreatectomies [51,52]. Baseline characteristics and treatment regimens are described in Table 2. Evaluation of risk of bias resulted in a low overall risk of bias in the trial of Antila et al. and some concerns in the trial of Laainen et al. Details can be found in the Supplementary Materials.

The 90-day mortality was reported in both trials without any incidents. Pooled analysis of POPF showed significantly less events in the glucocorticoid group when compared to placebo (OR 0.22, 95% CI: 0.07 to 0.77, I^2^ = 3%). There was no significant difference in PPH (OR 0.54, 95% CI: 0.14 to 2.03, I^2^ = NA), DGE (OR 0.54, 95% CI: 0.2 to 1.45, I^2^ = 0%) or intraabdominal fluid collections (OR 0.47, 95% CI: 0.15 to 1.52, I^2^ = 0%). LOS was equal in both groups (MD −0.63 days, 95% CI: −3.93 to 2.67, I^2^ = 0%). The certainty of evidence of all pooled outcomes of this analysis was very low.

Additionally, one trial evaluated pasireotide versus hydrocortisone after pancreatic surgery [46]. There was no significant difference in postoperative complications (MD CCI-score: −6.17; 95% CI: −12.81 to 0–47, *p* = 0.07), POPF (OR 1.39; 95% CI: 0.68 to 2.82, *p* = 0.37), and 30-day mortality rate (OR 2.0, 95% CI: 0.18 to 22.62, *p* = 0.58) in the pasireotide group versus the hydrocortisone group. Overall risk of bias was judged low.

### 3.4. Prokinetics

Two trials with a total of 146 patients were included for this analysis [6,53]. The trial of Ohwada et al. analyzed 31 patients after pylorus-preserving pancreatoduodenectomy [53]. Yeo et al. included a fast majority of pylorus-preserving duodenectomies (85%), but classic Whipple procedures and total pancreatectomies were also analyzed [6]. Treatment regimens and baseline characteristics are presented in Table 3. Overall risk of bias was high in the trial of Yeo et al. and some concerns were seen in the trial of Ohwada et al. Details on risk of bias assessment can be found in the Supplementary Materials.

Both trials reported on DGE and nasogastric tube removal. For both outcomes, there was no significant difference found (DGE: OR 0.33, 95% CI: 0.08 to 1.30, I^2^ = 0%, nasogastric tubes removal: MD 4.08 days, 95% CI: −10.74 to 2.58, I^2^ = 99%). The certainty of evidence was very low for both comparisons.

Gastric motility was reported differently in the included trials. Erythromycin non-significantly reduced the proportion of retention of liquids at 30 min (*p* = 0.07) and was associated with a significant improvement in solid emptying (20–120 min, NA, *p* < 0.01) when tested with radionucleotide gastric-emptying trials reported by Yeo et al.

Only one adverse effect described as abdominal cramping was reported in the trials of Ohwada et al. No major adverse reactions were attributed to erythromycin in the trials of Yeo et al. There was no significant difference in complication rate in the trial of Yeo et al. (27.6% vs. 36.7%, *p* = 0.635).

### 3.5. Proton Pump Inhibitor

Two trials were found that reported on PPI after pancreatic resections (Table 4) [7,9]. Both trials investigated patients after pylorus-preserving pancreatoduodenectomy and were judged with a high overall risk of bias. Pooling was not possible as the trials did not report on the same outcomes of interest.

The trial of Toyota et al. analyzed 24 patients that received 20 mg of dissolved Omeprazole through a jejunal feeding tube twice daily for 7 days after surgery [7]. The control group consisting of 18 patients received the same volume of saline as a placebo (six exclusions due to gastric bleeding). Baseline characteristics were equally distributed. Eight complications were reported in the PPI-Group whereas seventeen were reported in the control group. Notably, there were no gastric bleedings in the PPI group compared to six in the control group (*p* < 0.05). All other reported complications were equally distributed.

Jang et al. included 18 patients receiving 30 mg of lansoprazole daily for 12 weeks postoperatively [9]. A total of 19 patients served as comparisons in the control group where no additional treatment was reported. There was no difference in baseline characteristics and perioperative parameters.

During a modified Lundh meal test, gastrin serum concentrations were measured. Postoperative gastrin levels were approximately twice as high compared to preoperatively in the lansoprazole group (100 pg/mL vs. 50 pg/mL after 30 min, *p* < 0.001), whereas there was no difference in the control group. Pancreatic volume decreased significantly by 44.0% in the control group compared to 10.7% in the intervention group (95% CI: NA, *p* < 0.001). Nutritional status was reported by percentage of body weight lost after 3 months (4.5% vs. 9.9%, 95% CI: NA, *p* = 0.007), patients who regained 95% of their previous body weight (12 vs. 7 patients, *p* = 0.072) and mean triceps skin-fold thickness (−8.7% vs. −21.5%, 95% CI: NA, *p* = 0.047) in favor of the lansoprazole group.

Pancreatic endocrine insufficiency was defined as pancreoprivic diabetes or prediabetes (fasting plasma glucose 110–125 mg/dL and 2-h glucose level 140–199 mg/dL). This was the case in 3 out of 16 patients in the intervention group and 7 out of 15 in the control group. Additionally, serum insulin levels were higher in the lansoprazole group (21.1, SD 12.1 uU/mL, 101.0% of preoperative level) than in the control group (6.9 SD 1.6 uU/mL, 47.6% of preoperative level) 3 months postoperatively. Exocrine function showed a significantly lower level of stool elastase in the control group when compared to lansoprazole (24 (SD6) vs. 59 (12) ug/L, *p* = 0.009). Only pancreatic leakage was reported as a complication with no significant difference (4 (22.2%) vs. 3 (15.8%), *p* = 0.332).

### 3.6. Antidiabetic Drugs

Two trials were found that met the inclusion criteria (Table 5) [54,55]. Due to different treatments, pooling was not possible.

Patients with an artificial pancreas device (SGT-22; Nikkiso Company, Tokyo, Japan, *n =* 17) were compared to patients with a sliding scale method (*n =* 13) in the trial of Okabayashi et al. [54]. Thirty-day postoperative mortality was zero in both groups. Blood glucose levels were significantly different between 2 to 18 h after surgery comparing both groups with lower values in the artificial pancreas group compared to the sliding scale group (*p* < 0.05). Mean total insulin use was 107IU (SD 109) in the artificial pancreas group versus 8IU (SD 6) in the sliding scale group during the first 18 h after surgery (*p* < 0.001). Overall risk of bias was high due to some concerns in randomization, deviations from intended intervention, and outcome measurement.

Van Veldhuisen et al. performed a randomized cross-over analysis of a bihormonal artificial pancreas device with closed-loop glucose control (BIHAP) vs. current diabetes care after total pancreatectomy [55]. Time in euglycemia (70–180 mg/dL (3.9–10 mmol/L)) was significantly longer during treatment with BIHAP (median, 78.30%; IQR, 71.05–82.61%) than with current diabetes care (median, 57.38%; IQR, 52.38–81.35%; *p* = 0.03). Furthermore, the time spent in hypoglycemia (<70 mg/dL (3.9 mmol/L)) was lower with BIHAP (median, 0.00% (IQR, 0.00–0.07%) vs. 1.61% (IQR, 0.80–3.81%); *p* = 0.004) in the control group. No serious adverse events were observed during the trial period of fourteen days. The trial was rated with some concerns on bias due to outcome measurements and low concerns for all other assessments.

### 3.7. Pancreatic Enzyme Replacement Therapy

There were eight trials reporting on PERT that met our inclusion criteria (Table 6) [56,57,58,59,60,61,62,63]. Study protocols were not comparable. Therefore, pooling was not possible. The intervention group was defined as PERT when compared to placebo or high dose PERT when compared to low dose. Risk of bias assessment resulted in low risk of bias in one trial, some concerns in five trials, and a high risk of bias in two trials.

Kim et al. included patients with a stool elastase level < 200 ug/g. The intervention group received 40,000 IU 3 times daily for 3 months beginning at the first outpatient follow up, usually 3–4 weeks after surgery (*n =* 118). The control group received a placebo capsule comprising lactose and cellulose instead (*n =* 119). Except for weight, the baseline characteristics were equally distributed. Compliance was 69.1% in the intention to treat analysis.

After 3 months of therapy or placebo, the difference in weight change did not reach statistical significance (−0.68 kg for the PERT group vs. −1.19 kg for the placebo group; *p* = 0.31) in the intention to treat analysis. Stool frequency was equal in both groups (1.2 vs. 1.3, MD −0.10, *p* = 0.13). Stool elastase was 41.7 in the intervention group and 57.2 in the placebo group (MD −15.5, 95%CI: −33.6–2.6, *p* = 0.09). Prealbumin as a marker for nutritional status did reach statistical significancy in the intention to treat analysis (24.3 vs. 21.9, MD 2.4, 95%CI: 0.79–4.01, *p* = 0.003). In a multivariate analysis, high preoperative BMI as well as poor compliance were risk factors for postoperative weight loss.

The trial protocol of Seiler et al. included one week of no PERT before the baseline measurements in patients with CFA (coefficient of fat absorption) < 80%. In the double-blind phase of 7 days, the participants received either 25,000 international units (IE) Creon (*n =* 31) or placebo (*n =* 25) for 9–15 capsules daily. Major protocol deviation such as lack of compliance was seen in 20.7%. There was no difference in overall complication rate (13.6% vs. 16.8%, *p* = 0.486).

After the double-blind phase, CFA was 21.4 vs. −4.2 (MD 25.6 95% CI: 13.9–37.3, *p* < 0.001), coefficient of nitrogen absorption (18.9 vs. −10.3, MD 29.2, 95% CI 16.7–41.8, *p* < 0.001), and stool fat g/day −24.0 vs. 6.1 (MD 30.2, 95% CI 40.2–20.1, *p* < 0.001). Stool weight was 282 g/d vs. 514 g/d (MD −232, 95% CI −348.8–−115.2, *p* < 0.001). Stool frequency per day was 1.6 vs. 2.8 (MD −1.2, 95% CI; −1.9–0.48, *p* = 0.001).

Neoptolemos included 35 patients for the final analysis of a cross-over study. High dose pancreatin was compared to standard dose pancreatin after a run-in period of 2 weeks and then for two 14 day periods. Results were taken at the end of each period. Results of both groups were summarized for final analysis (high dose 1 + high dose 2 and low dose 1 + low dose 2). Details can be seen in the Supplementary Materials.

There was no difference in daily stool-fat excretion between high and standard dose pancreatin (12.0 vs. 11.1, MD 0.9, 95% CI −2.78–4.58, *p* = 0.63) or stool volume (575 g/d vs. 536 g/d, MD 39, 95% CI −90.5–168.5, *p* = 0.55. Stool frequency was equal in both groups (1.6 vs. 1.6).

Finally, the study group of van Hoozen et al. analyzed 11 patients after pancreatic resections (local resection—longitudinal pancreatojejunostomy) due to chronic pancreatitis. After a 4-week period where both groups received pancreatin, pancreatin therapy was either continued (*n =* 5) or replaced by placebo (*n =* 6) for another 4 weeks.

Comparing treatment outcomes at 8 weeks, patients randomized to receive placebo demonstrated significantly worse fat and total energy absorption than patients who continued to receive pancreatin supplementation (*p* < 0.02 and *p* < 0.02). No differences in stool frequency were seen in this trial.

## 4. Discussion

This systematic review and meta-analysis of perioperative medical management in pancreatic surgery shows less pancreatic fistulas when using somatostatin analogs and for glucocorticoids perioperatively. This result was, however, observed only with a moderate and very low certainty of evidence according to the GRADE Criteria. For other medical interventions, the evidence from randomized clinical trials is limited. The above analyzed medical interventions are discussed separately in the following passages. A regularly updated living meta-analysis can be found on www.emps.evidencemap.surgery (accessed on 3 January 2023) [2].

### 4.1. Somatostatin Analogues

Octreotide and other somatostatin analogues reduce the secretion of pancreatic enzymes through two different mechanisms [64]. The exocrine pancreas function is inhibited by the direct inhibition of pancreatic acinar cells as well as indirect inhibition through other pancreas-stimulating hormones such as gastrin. Less flow of digesting pancreatic enzymes through the pancreatic anastomosis after PD or less pressure at the distal end after DP through less pancreatic fluid production are thought to have a beneficial influence on healing [65].

Often-used medicaments in the included studies are octreotide and somatostatin. Somatostatin due to its short half-life time must be infused continuously whereas octreotide can be administered subcutaneously every eight hours [64]. Most of the included studies did administer somatostatin analogues postoperatively for seven days. In line with this regimen, the effect of somatostatin analogues decreases after seven days through adaption, desensitization, and tachyphylaxis [66].

In the present meta-analysis, somatostatin analogues did reduce POPF with a low certainty of evidence. This was shown in both PD and DP and may lead to a shorter hospital stay. This did, however, not influence mortality or other complications such as PPH, DGE, bile leak, or intraabdominal abscesses.

Limitations of this pooled analysis must be considered in the heterogeneity of treatment regimes. Octreotide was the most commonly used analogue but also somatostatin, pasireotide, and vapreotide were administered. Three studies administered an additional preoperative dose [31,39,44]. These studies, however, did not show a benefit of preoperative somatostatin administration when analyzed individually. One trial included six patients after TP [19]. Exclusion of this trial for POPF analysis did not relevantly affect the overall effects. Furthermore, definition of POPF has changed recently [14]. Pancreatic fistulas grade A, now defined as biochemical leaks, were not included in this analysis when reported in the included trials but were not always distinguishable as such. However, a sub-analysis on POPF Grade B and C according to the ISGPS definition confirmed a benefit of using somatostatin analogues [14]. Last, as shown in multiple studies, a surgeon’s experience, and the treatment volume of the clinic have a relevant impact on surgical outcomes [67]. The heterogeneity of included patients in the analyzed trials may implicate different surgical volumes. The effect of additional treatment may be influenced by the a priori risk of development of POPF. The risks of POPF are higher with soft pancreatic texture and main pancreatic duct diameter ≤ 3 mm [68]. Common adverse effects include nausea, abdominal cramps, or diarrhea and can be treated adequately [69].

Therefore, the evidence for routine usage of somatostatin postoperatively is limited. Further research evaluating the effectiveness of somatostatin analogues in specific cohorts such as patients at high risk for POPF undergoing PD are needed. The new definition of the ISGPS creating risk classes should be used for such research [68].

### 4.2. Glucocorticoids

A possible cause for postoperative complications such as POPF and DGE after partial pancreatectomies may be postoperative pancreatic inflammation, mainly caused by pancreatic damage due to surgical manipulation at the transection line [52]. Animal models have shown that acinar cells are relevant in this inflammation cascade and that the severity of postoperative pancreatitis is reduced by perioperative glucocorticoids [70,71]. Both analyzed trials included only patients with high acinar-cell-rich transection lines, thus those groups of patients in whom the greatest effect is expected. The inflammation process at the transection line peaks within 4 h of resection and causes edema and activation of pancreatic enzymes compressing healing and duct obstruction [72]. Additionally, a retrospective analysis showed better survival rates when dexamethasone was administered perioperatively. However, there was no reporting on postoperative complications in that trial [73]. Complications often feared in the use of glucocorticoids are infectious complications and decreased anastomotic and wound healing. However, a meta-analysis on the use of perioperative glucocorticoids in oncologic abdominal surgery showed lower postoperative IL6 (POD1) and CRP (POD 3) values and additionally less postoperative infectious complications [74]. Anastomotic leakage was equal in both groups of the mentioned meta-analysis where, due to the publication date, the discussed pancreatic trials were not included. In accordance with the findings in the meta-analysis, there was no significant difference in infectious complications, wound healing, DGE, PPH, mortality, and LOS in both trials. Antila et al. showed a decreased rate in clinically relevant POPF (Grade B + C) in patients after distal pancreatectomy. The difference in POPF for patients after PD in the trial of Laainen et al. was insignificant. The evidence of these findings is however very low and limited due to the small sample sizes of the included studies. Further trials are needed to investigate the role of glucocorticoids in pancreatic surgery.

### 4.3. Erythromycin

Delayed gastric emptying after pylorus-preserving pancreatoduodenectomies is thought to have different mechanisms. Peritonitis from anastomotic leakages, ischemia of the pyloric muscle cells, damage to the vagal nerve, and reduced circulating motilin levels are discussed [75]. Furthermore, opioids that are often prescribed directly after pancreatic surgery for pain management increases severity of DGE [76]. In 2007, and therefore after the included studies were published, the ISGPS developed a standardized definition of DGE based on time to tolerance of solid food intake and nasogastric tube necessity [48]. Erythromycin, primarily invented as an antibiotic medicament, showed agonistic effects on the motilin receptor and therefore has a beneficial effect on resolving gastroparesis [77]. Erythromycin is associated with QT prolongation and interactions with other agents that are metabolized by CYP3A4 [77]. Due to different treatment protocols, pooling of the included studies was not possible. Erythromycin did reduce DGE in the trial of Ohwada et al. [53] and non-significantly in the trial of Yeo et al. [6]. However, certainty of evidence on earlier nasogastric tube removal and quicker progression to diet is low [53].

These results must be interpreted with caution as sample sizes were small and limited data exist overall for the efficacy of erythromycin in patients with gastroparesis. However, when DGE is present, erythromycin may be a valuable option.

### 4.4. Proton Pump Inhibitor

PPI inhibit hydrogen-potassium ATPase of gastric parietal cells and therefore lower gastric acid production [78]. This has shown to be prophylactic for gastric bleeding in the trial of Toyota et al. [7]. Not only acute bleeding but also the rate of marginal ulcers at the gastrojejunostomy can be reduced by antisecretory drugs such as H_2_ -Receptor inhibitors and PPI [79]. Chronic irritation of the anastomosed jejunum due to the acidic environment, which it is worse at buffering than the duodenum due to lacking Brunner glands, could also lead to cancer formation at the anastomotic site [80]. For these reasons, PPI are routinely prescribed worldwide after PD [79].

An additional effect of PPI is drug-induced hypergastrinemia, a trophic stimulator of exocrine pancreatic cells [78]. In animal models, this has shown to be preventive for pancreatic atrophy [81,82]. These findings could also be shown in the trial of Jang et al. with less pancreatic atrophy and a better exocrine (stool elastase) and endocrine (insulin levels) function postoperatively [9]. The authors concluded that this had a decisive effect on a better nutritional status and less weight loss. Most results, however, failed to show a significant difference which may be due to the trials being underpowered.

Due to the prevention of anastomotic bleeding, postoperative administration of PPI after pancreatoduodenectomy should be considered.

### 4.5. Antidiabetic Drugs

Partial pancreatic resections pose a significant risk for endocrine pancreatic insufficiency as approximately one out of five patients will develop pancreatic endocrine insufficiency after PD or DP according to long-term evaluations [83]. With total pancreatic resection, insulin management is even more delicate due to the abundance of the peptide hormone insulin that is produced in the pancreatic beta-cells of the islets of Langerhans and the opposing hormone glucagon. This leads to significant morbidity and mortality [84].

Furthermore, hyperglycemia induced by surgical stress is thought to impair postoperative recovery [85]. The studies of Okabayashi and van Veldhuizen et al. showed better postoperative blood glucose control and no serious adverse events with closed loop systems [54,55]. However, long-term outcomes are lacking.

More research is needed for the development of new artificial pancreatic devices to avoid the long-term consequences of hyperglycemia and to impede the risk of hyperglycemia. Regardless to this, regular follow-ups for endocrine pancreatic insufficiency and consultation of an endocrinologist if endocrine pancreatic insufficiency is present is recommended.

### 4.6. Pancreatic Enzyme Replacement Therapy

After pancreatic resections, exocrine function may be impaired leading to steatorrhea and intestinal malabsorption [8]. Several trials did compare pancreatic enzymes to placebo or high dose versus low dose PERT. Intestinal fat absorption was significantly better in the PERT groups when compared to placebo [60,61]. Compliance did have a significant effect on weight loss [57]. To improve compliance, high dose PERT-capsules have been introduced to the market to lessen the capsules needed per day. However, there is no evidence on the benefit of high versus low dose pancreatin replacement as stated by Neoptolemus et al. [58].

These results indicate an advantage in intestinal absorption. Intestinal absorption may be even more important in patients with pancreatic surgery who might suffer from malnutrition due to pre-existing pancreatic exocrine insufficiency caused by chronic pancreatitis or who might have had significant weight loss due to oncological reasons [86]. Therefore, a patient-adjusted dose of PERT treatment should be initiated after pancreatic resections. The focus should be on improving compliance to therapy.

## 5. Conclusions

Many trials on treatment with somatostatin analogues were found and there is a potential reduction in POPF. However, further research evaluating the effectiveness of somatostatin analogues in specific cohorts such as patients at high risk for POPF undergoing PD are needed. Data on glucocorticoids and its role in reducing complications after pancreatic surgery are limited. Further studies are needed to confirm the potential benefit for POPF reduction. There is low evidence for the use of erythromycin and proton pump inhibitors. Trials on antidiabetic drug treatment to provide glucose control after pancreatic surgery show promising results but are limited to a few trials. PERT improves intestinal fat absorption and is recommended to avoid malassimilation because of exocrine pancreatic insufficiency.

## Figures and Tables

**Table 1:** Somatostatin and analogues.

First Author	Year	Country	Treatment	Primary Outcome	Secondary Outcome	Patient Randomized	Patients Analyzed	Pathology	Surgical Approach
Cao [23]	2021	China	Somatostatin vs. Placebo	POPF	Biochemical leak, morbidity, pancreatectomy-related complications	205	199	PDAC or pancreatitis *n =* 90 Other diseases *n* = 109	Open PD *n =* 127 Laparoscopic PD *n =* 72
Tarvainen [46]	2020	Finland	Hydrocortisone vs. Pasireotide	Comprehensive Complication Index (CCI) score within 30 days	Clavien–Dindo classification	168	126	PDAC *n* = 27 Cholangiocarcinoma *n =* 9 IPMN *n =* 12 MCN *n =* 8 PNET *n =* 27 Serous cystadenoma *n =* 4 Papilla adenoma *n =* 3 Dysplasia *n =* 11 Metastasis of another carcinoma *n =* 5	DP *n =* 57 PD *n =* 60 Papillectomy *n =* 1 Enucleation *n =* 6
Kriger [47]	2020	Russia	somatostatin analogues and glucocorticoids vs. somatostatin analogue	POPF	N/A	78	78	N/A	N/A
You [45]	2019	Korea	Octreotide vs. Placebo	Pancreatic juice output	Incidence of POPF and postoperative complications	66	59	Bile duct cancer 24 Pancreatic cancer 17 Ampullary cancer 11 Others 5	PD *n =* 59
El Nakeeb [25]	2018	Egypt	Octreotide vs. Placebo	POPF (period: 30 days after surgery)	DGE [48], length of hospital stay	104	104	Adenocarcinoma *n =* 89 Neuroendocrine *n =* 1 Cholangiocarcinoma *n =* 2 Solid pseudopapillary tumor *n =* 3 Adenoma *n =* 5 Pancreatitis *n =* 3 Benign cyst *n =* 1	PD *n =* 104
Kurumboor [34]	2015	India	Octreotide vs. Placebo	POPF (period: 30 days after surgery)	Postoperative complications	109	109	Soft pancreas Non-dilated ducts	PD *n =* 109
Kong [49]	2016	China	Octreotide vs. Placebo	POPF	Hospitalization days, treatment cost	306	306	N/A	N/A
Allen [17]	2014	USA	Pasireotide vs. Placebo	60-day ≥grade 3 pancreatic complication rates (fistula, leak, and abscess)	60-day: overall complication rate, mortality, pancreatic complication rate; Amylase level; duration of drainage, daily drain volume, time of return of bowel function as defined by passage of flatus	443	300	PDAC *n =* 154	PD *n =* 220 DP *n =* 80
Belyaev [19]	2013	Germany	Octreotide (intra-arterial)	Increased pancreatic hardness	POPF, DGE	26	25	PDAC *n =* 14 Ampullary cancer *n =* 1 Distal hepatic duct cancer *n =* 1 Duodenal cancer *n =* 1 Melanoma metastasis *n =* 1 Benign *n =* 7 Chronic pancreatitis *n =* 3 Pseudocyst *n =* 1 IPMN *n =* 1 Cystadenoma *n =* 1 Duodenal adenoma *n =* 1	PD *n =* 19 TP *n =* 6
Fernandez-Cruz [27]	2013	Spain	Octreotide vs. placebo	POPF	Morbidity, hospital mortality and duration of postoperative hospital length of stay	62	62	PDAC *n =* 32 Ampullary carcinoma *n =* 10 IPMN *n =* 5 Cholangiocarcinoma *n =* 4 Neuroendocrine tumor *n =* 3 Metastatic tumor *n =* 2 Duodenal cancer *n =* 2 Pseudopapillary solid tumor *n =* 2 Serous cystadenoma *n =* 2	PD *n =* 62
Wang [43]	2013	China	Somatostatin vs. Placebo	POPF	Postoperative complications	38	38	Pancreatic neoplasm *n =* 21 Chronic pancreatitis *n =* 14 CBDC *n =* 4 Benign pancreatic cancer *n =* 11 Duodenal cancer 10	PD
Katsourakis [31]	2010	Greece	Somatostatin vs. Placebo	Effect of somatostatin administration on the ultra-structure of exocrine pancreatic cells	Postoperative complications	67	67	PDAC *n =* 53 Pancreatitis *n =* 9 Neuroendocrine tumor *n =* 1 Metastatic adenocarcinoma *n =* 1 Lymphoma *n =* 1 Acinar cell *n =* 1 Cystadenoma *n =* 1	PD *n =* 59 DP + splenectomy *n =* 7 DP *n =* 1
Kollmar [33]	2008	Germany	Somatostatin vs. Placebo	Incidence DGE	Perioperative morbidity other than DGE	67	67	N/A	PD *n =* 67
Closset [24]	2008	Belgium	Somatostatin vs. Octreotide	Pancreatic stump-related complications	-	50	50	IPMT *n =* 11 Ampulloma *n =* 7 Serous cystadenoma *n =* 2 GIST *n =* 1 Endocrine tumor *n =* 2 Duodenal tumor *n =* 1 Cholangiocarcinoma *n =* 1 PAN IN 3: *n =* 1	PD *n =* 50
Ramos-De la Medina [50]	2006	USA	Vapreotide vs. placebo	Pancreas-specific complications; mortality	Overall complications; duration of hospitalization	381	275	Benign neoplasm *n =* 41 PDAC *n =* 80 Ampullary carcinoma *n =* 22 Duodenal carcinoma *n =* 6 Bile duct carcinoma *n =* 8 IPMN *n =* 1 Neuroendocrine *n =* 14 Cystadenocarcinoma *n =* 1 Other *n =* 7	DP *n =* 38 PD *n =* 98 PPPD *n =* 58
Hesse [30]	2005	Belgium	Low-dose Octreotide vs. Placebo	General complications, including extended length of hospital stay	N/A	105	105	Cancer *n =* 80 Benign tumor *n =* 8 Chronic pancreatitis	PD *n =* 80 LPJ= 12 DP = 10 CJ = 3
Suc [41]	2004	France	Octreotide vs. Placebo	IACs	EACs isolated or associated with IACs	230	230	N/A	N/A
Sarr [39]	2003	USA	Somatostatin analogue: vapreotide vs. Placebo	Development of pancreatic-related complications	Overall complication rate	275	275	Benign neoplasm *n =* 44 Malignant *n =* 138 IPMN *n =* 1 Neuroendocrine *n =* 14 Cystadenocarcinoma *n =* 1 Other *n =* 7	PD *n =* 134 PPPD *n =* 80 DP *n =* 52 Central pancreatectomy *n =* 8
Shan [40]	2003	Taiwan	Somatostatin vs. Placebo	Prevention of pancreatic stump-related complications	N/A	60	54	Pancreatic cancer *n =* 12 Distal CBD cancer *n =* 7 Ampullary cancer *n =* 15 Duodenal cancer *n =* 4 Duodenal stromal cancer *n =* 4 Benign lesion of periampullary area *n =* 6 Lymphoma *n =* 3 Other malignancy *n =* 3	Whipple *n =* 31 PPPD *n =* 24
Falconi [26]	2002	Italy	Lanreotide vs. Placebo	Exocrine pancreatic secretion	N/A	8	7	PDAC *n =* 3 Periampullary cancer *n =* 2 Duodenal carcinoma *n =* 1 Cystic carcinoma *n =* 1 Neuroendrocrine tumor *n =* 1	PPPD
Gouillat [29]	2001	France	Somatostatin vs. Placebo	Reduction in pancreatic juice outcome	Amylase and lipase output	75	75	PDAC *n =* 61 Chronic pancreatitis *n =* 4 Other tumors *n =* 10	PD = 38 PPPD *n =* 37
Bonora [20]	2001	Italy	Gabexate mesilate vs Gabexate mesilate combined with Octreotide	Postoperative complications	-	50	50	PDAC *n =* 15 Periampullary carcinoma *n =* 5 Duodenal carcinoma *n =* 1 Endocrine neoplasm *n =* 6	DPPHR PD Enucleation
Yeo [44]	2000	USA	Octreotide vs. Saline (Placebo)	POPF, total complications, death, and length of hospital stay	Cost of octreotide and the potential cost savings associated with the cessation of its use	383	211	PDAC *n =* 84 Bile duct carcinoma *n =* 37 Ampullary carcinoma *n =* 27 Chronic pancreatitis *n =* 22 Islet cell tumor *n =* 9 Periampullary adenoma *n =* 8 Duodenal adenocarcinoma *n =* 4 Gastrointestinal stromal tumor *n =* 2	PD
Briceno Delgado [2]	1998	Spain	Octreotide	POPF	Overall postoperative complications	34	34	Ampulloma *n =* 17 Pancreatic cancer *n =* 8 Cholangiocarcinoma *n =* 2 Duodenal carcinoma *n =* 1 Chronic pancreatitis *n =* 5	PD *n =* 34
Lowy [36]	1997	USA	Octreotide	Development of a clinical or biochemical pancreatic anastomotic leak	Gastrointestinal function	120	110	PDAC *n =* 64 Periampullary adenocarcinoma *n =* 20 Neuroendocrine tumor *n =* 9 Other malignant tumor *n =* 12 Benign *n =* 5	PD
Friess [28]	1995	Switzerland	Octreotide	Postoperative complications	-	247	247	Chronic pancreatitis	PD *n =* 70 DP *n =* 55 PPPD *n =* 54 PJ *n =* 61 Other *n =* 7
Beguiristain [18]	1995	Spain	Somatostatin	POPF	Postoperative complications	35	35	Periampullary cancer *n =* 14 Pancreatic cancer *n =* 13 Chronic pancreatitis *n =* 3 Endocrine tumor *n =* 1 Gastric cancer *n =* 1 Cystadenoma *n =* 1	PD
Montorsi [37]	1995	Italy	Octreotide vs. Placebo	POPF	Other postoperative complications	218	218	Pancreatic and periampullary cancer *n* = 139 Other abdominal neoplasm *n =* 37 Chronic pancreatitis *n =* 18 Endocrine tumor *n =* 14 Miscellaneous *n =* 8	PD *n =* 143 LR *n =* 54 SP *n =* 12 Enucleation *n =* 5 Other *n =* 4
Pederzoli [38]	1994	Italy	Octreotide	Postoperative complications	-	303	252	PDAC *n =* 61 Periampullary tumor *n =* 43 Endocrine tumor *n =* 24 Cystic tumor *n =* 24 Chronic pancreatitis *n =* 95 Other *n =* 5	Whipple *n =* 100 DPPHR *n =* 5 DP *n =* 60 Intermediate resection *n =* 7 Enucleation *n =* 14 PJ *n =* 66
Tulassay [42]	1993	Hungary	Somatostatin	Postop. increase in pancreatic enzymes	-	33	33	Cyst of pancreas *n =* 19 Chronic pancreatitis *n =* 14	Cysto-duodenostomy *n =* 12 Cysto-gastrostomy *n =* 7 Wirsungo-gastrostomy *n =* 7 Wirsungoplastic *n =* 7
Büchler [22]	1992	Germany	Octreotide	Pancreatic fistula.	Abscess, acute pancreatitis, pulmonary insufficiency, shock, and sepsis, which represent local and systemic sequelae of a pancreatic leak	322	N/A	PDAC *n =* 71 Periampullary cancer *n =* 40 Endocrine tumor *n =* 9 Chronic pancreatitis *n =* 112 Others *n =* 14	DPPHR *n =* 48 Whipple *n =* 152 DP *n =* 31 PJ *n =* 8 Enucleation *n =* 3 Others *n =* 4
Lange [35]	1992	USA	Somatostatin vs. Placebo	Reducing pancreatic drainage	Postoperative complications	21	21	Gastrinoma *n =* 7 Insulinoma *n =* 14	Enucleation *n =* 10 Resection *n =* 11
Buccoliero [21]	1992	Italy	Somatostatin	Volume, pancreatic, and gall bladder secretion	Amylase and Lipase secretion, concentration of bicarbonates and chlorides	31	31	N/A	PD
Klempa [32]	1991	Germany	Somatostatin	Pancreatic juice: volume, amylase, lipase, protein and bicarbonate	Pancreatic exocrine function	30	30	Pancreas carcinoma *n =* 19 Ampullary carcinoma *n =* 5	PD

PDAC: Pancreatic ductal adenocarcinoma; IPMN: Intraductal Papillary Mucinous Neoplasm; pNET: Pancreatic neuroendocrine tumors; PAN IN 3: pancreatic intraepithelial neoplasia 3 PD: Pancreatoduodenectomy; DP: distal pancreatectomy; DPPHR: Duodenum-preserving pancreatic head resection; PPPD: Pylorus-preserving pancreatoduodenectomy PJ: Pancreatojejunostomy; LR: left resection; SP: Subtotal pancreatectomy; LPJ: Longitudinal pancreatic jejunostomy, CJ: cysto-jejunostomy. POPF: Postoperative Pancreatic Fistula.

**Table 2:** Corticosteroids.

First Author	Year	Country	Treatment	Primary Outcome	Secondary Outcome	Patient Randomized	Patients Analyzed	Pathology	Surgical Approach
Tarvainen [46]	2020	Finland	Hydrocortisone vs. Pasireotide	Comprehensive Complication Index (CCI) score within 30 days	POPF, DGE, PPH, readmissions, all within 30 days after the operation, length of hospital stay	168	126	PDAC *n =* 27 Cholangiocarcinoma *n =* 9 IPMN *n =* 12 MCN *n =* 8 pNET *n =* 27 Serous cystadenoma *n =* 4 Papilla adenoma *n =* 3 Dysplasia *n =* 11 Metastasis of another carcinoma *n =* 5	DP *n =* 57 PD *n =* 60 Papillectomy *n =* 1 Enucleation *n =* 6
Antila [51]	2019	Finland	Hydrocortisone vs. Placebo	Overall complications (C-D III-V)	Clinically significant POPF	40	31	PDAC *n =* 5 pNET *n =* 4 Cystic tumor *n =* 17 Other *n =* 2	DP
Laaninen [52]	2016	Finland	Hydrocortisone vs. Placebo	Urine trypsinogen positive days, overall complications (Clavien–Dindo III-IV).	Clinically pancreatoduodenectomy-related complications (POPF, PPH, DGE; grades B and C), mortality, and general infectious complications	155	62	PDAC *n =* 27 BDC *n =* 14 IPMN *n =* 5 Duodenal carcinoma *n =* 7 Gastrointestinal stromal tumor *n* = 3 Chronic pancreatitis *n =* 3 other = 3	

PDAC: Pancreatic ductal adenocarcinoma; IPMN: Intraductal Papillary Mucinous Neoplasm; MCN: Pancreatic mucinous cystic neoplasm; pNET: Pancreatic neuroendocrine tumors; BDC: bile duct carcinoma; PD: Pancreatoduodenectomy; DP: distal pancreatectomy; TP: Total pancreatectomy.

**Table 3:** Prokinetic Drug Treatment.

First Author	Year	Country	Treatment	Primary Outcome	Secondary Outcome	Patient Randomized	Patients Analyzed	Pathology	Surgical Approach
Ohwada [53]	2001	Japan	Erythromycin vs. Placebo	DGE	Gastric motility Nasogastric Tube Removal	31	31	PDAC *n =* 4 Bile duct carcinoma *n =* 8 Ampullary tumor *n =* 11 Duodenal tumor *n =* 2 Chronic pancreatitis *n =* 3	PPPD
Yeo [6]	1993	USA	Erythromycin vs. Placebo	DGE	N/A	118	118	Pancreas cancer *n =* 78 Bile duct carcinoma *n =* 12 Ampulla *n =* 17 Duodenum *n =* 11	TP *n =* 3 Partial pancreatectomy *n =* 115

PDAC: Pancreatic ductal adenocarcinoma; PPPD: pylorus-preserving pancreatoduodenectomy; TP: total pancreatectomy.

**Table 4:** Proton-Pump-Inhibition.

First Author	Year	Country	Treatment	Primary Outcome	Secondary Outcome	Patient Randomized	Patients Analyzed	Pathology	Surgical Approach
Jang [9]	2003	Korea	Lansoprazole (PPI) vs. Placebo	Hypergastrinemia, volume of the distal pancreas, nutritional status, endocrine and exocrine function,	Serum gastrin levels before surgery and 3 months after surgery.	56	37	Ampullary cancer *n =* 16 Bile duct cancer *n =* 13 PDAC *n =* 6 Duodenal cancer *n =* 2	PD
Toyota [7]	1998	Japan	Omeprazole (PPI) vs. Placebo	Gastric stasis	Volume and acidity of the gastric juice	42	42	Bile duct cancer *n =* 7 Gallbladder cancer *n =* 1 Pancreatic head carcinoma *n =* 19 Papilla of Vater Cancer *n =* 5 Chronic pancreatitis *n =* 7 Papillitis *n =* 2 Congenital biliary dilatation *n =* 1	PPPD

PD: Pancreatoduodenectomy; PPPD: pylorus-preserving pancreatoduodenectomy.

**Table 5:** Antidiabetic Drug Treatment.

First Author	Year	Country	Treatment	Primary Outcome	Secondary Outcome	Patient Randomized	Patients Analyzed	Pathology	Surgical Approach
Van Veldhuisen [55]	2022	Netherland	Closed-Loop Glucose Control vs Current Diabetes Care	Median percentage of time spent in euglycemia	Safety and efficacy of the BIHAP	12	10	IPMN *n =* 1 Benign *n =* 3 Malignant *n =* 6	TP
Okayabashi [54]	2009	Japan	Glucose Control by Artificial Pancreas	Incidence of severe hypoglycemia (40 mg/dL)	Total amount of insulin required for glycemic control in the first 18 h after pancreatic resection	32	30	Pancreatic disease	PD *n =* 15 DP *n =* 11 TP *n =* 2

IPMN: Intraductal Papillary Mucinous Neoplasm; PD: Pancreatoduodenectomy; DP: distal pancreatectomy; TP: total pancreatectomy.

**Table 6:** Pancreatic Enzyme Replacement Therapy.

First Author	Year	Country	Treatment	Primary Outcome	Secondary Outcome	Patient Randomized	Patients Analyzed	Inclusion Criteria	Exclusion Criteria
Kim [57]	2020	Korea	PERT vs. Placebo	Change in body weight	Changes in bowel habits, nutritional parameters, and QoL	237	164	PDAC *n =* 103 AoVC *n =* 50 CBDC *n =* 36 IPMN. *n =* 14 PNET *n =* 10 Other *n =* 24	PD *n =* 40 PPPD *n =* 197
Yasukawa [63]	2020	Japan	PERT vs. Placebo	NAFLD within 1 year	Incidences of NAFLD at 1, 3, 6, and 12 months, the rate of improvement in NAFLD with high-dose transfer in the control group	84	80	PDAC 35 Bile duct cancer 25 Ampullar PNT 8 IPMN 5	PD *n =* 80
Satoi [59]	2016	Japan	PERT vs. Placebo	Frequency of NAFLD-development within 12 months after starting treatment	Postoperative exocrine and endocrine pancreatic insufficiency; BMI, serum albumin level, nutritional status	57	57	PDAC 53 IPMC 4	PD
Seiler [60]	2013	Germany	PERT	Mean CFA change from baseline to end of double-blind treatment	Stool frequency, body weight and BMI	58	51	Malignancy *n =* 14 Chronic pancreatitis *n =* 44	PD/PPPD *n =* 29 DPPHR *n =* 13 Other *n =* 12
Farkas [56]	2001	Hungary	PERT vs. Placebo	Exocrine function via fecal elastase determinations, amylum tolerance test, checks on the symptoms of maldigestion	N/A	40	40	Chronic pancreatitis *n =* 40	Partial and total pancreatectomy *n =* 14
Whitcomb [62]	2010	USA	PERT vs. Placebo	Coefficient of fat absorption (CFA)	Coefficient of nitrogen absorption (CNA), clinical symptoms, and safety parameters	54	53	N/A	N/A
Neoptolemos [58]	1999	Great Britain	PERT vs. Placebo	Stool fat (as g/d)	Stool volume (mL/d), clinical global impression of disease symptoms, diary card recordings, and patient’s treatment preference	39	36	Chronic pancreatitis 17 Necrotizing pancreatitis	PPPD
Van Hoozen [61]	1997	Nether-lands	PERT vs. Placebo	Nutritional status and intestinal absorption	N/A	11	11	Chronic pancreatitis	LR-LPJ

PDAC: Pancreatic ductal adenocarcinoma; IPMN: Intraductal Papillary Mucinous Neoplasm; MCN: Pancreatic mucinous cystic neoplasm; pNET: Pancreatic neuroendocrine tumors. AoVC: Ampullar of Vater Cancer; CBDC: common bile duct cancer BDC: bile duct carcinoma; PD: Pancreatoduodenectomy; DP: distal pancreatectomy; TP: Total pancreatectomy; PPPD: pylorus-preserving pancreatoduodenectomy: DPPHR: duodenum-preserving pancreatic head resection; LR: left resection/distal pancreatectomy.

## Data Availability

All data is available at https://www.evidencemap.surgery/, accessed on 3 January 2023.

## Supplementary Materials

The following supporting information can be downloaded at: https://www.mdpi.com/article/10.3390/jcm12051750/s1.
