# Supplementary material for: Perioperative Drug Treatment in Pancreatic Surgery—A Systematic Review and Meta-Analysis
Source: J Clin Med. 2023 Feb 22;12(5):1750. doi: 10.3390/jcm12051750 (PMC10003556; doi:10.3390/jcm12051750)
Supplement: Supplementary file 1 [file jcm-12-01750-s001.zip › jcm-2174115-supplementary.pdf]

Supplementary material Perioperative Drug Treatment in Pancreatic Surgery – a systematic review and meta-analysis

Search strategy Embase

S1	pancrea* OR emb(pancreas)	579220*
S2	(resection* OR surger* OR surgical OR laparotom* OR enucleation* OR operation* OR operated) OR (pancreaticoduodenectom* OR pancreatoduodenectom* OR pancreatoduodenectom* OR duodenopancreatectom* OR pancreatectom* OR Whipple OR Kausch-Whipple OR ppWhipple OR dpphr OR PPPD)	9714270*
S3	(drug* OR medical*)	20559452*
S4	S3 AND S2 AND S1	146208*
S5	emb("pancreas surgery") LNK "drug therapy"	1075°
S6	emb(pancreaticoduodenectomy) LNK "drug therapy"	3917°
S7	emb(pancreatectomy) LNK "drug therapy"	1405°
S8	S4 OR S5 OR S6 OR S7	146853*

Search strategy Pubmed

((pancreas[MeSH terms] OR pancreas[tiab] OR pancreatic[tiab] OR pancreato*[tiab]) AND (resection* [tiab] OR removal [tiab] OR surger* [tiab] OR surgical [tiab] OR laparotom*[tiab] OR enucleation* [tiab] OR operation* [tiab] OR operated [tiab] OR 'surgical procedures, operative'[MeSH terms] OR 'general surgery'[MeSH terms])) OR (pancreaticoduodenectom*[tiab] OR pancreatoduodenectom*[tiab] OR pancreatoduodenectom*[tiab] OR duodenopancreatectom*[tiab] OR pancreatectom*[tiab] OR Whipple[tiab] OR Kausch-Whipple[tiab] OR ppWhipple[tiab] OR dpphr[tiab] OR PPPD[tiab] OR pancreaticoduodenectomy[MeSH] OR pancreatectomy[MeSH] OR 'Pancreas/surgery'[Mesh] OR 'Pancreatic Diseases/surgery'[Mesh]) AND (randomized controlled trial [pt] OR random*[tw] OR RCT [tw] OR 'Randomized Controlled Trials as Topic'[Mesh] OR 'Controlled Clinical Trial' [pt] OR systematic review [pt] OR meta-analysis [pt] OR review [pt] OR meta-analysis [tw] OR review [tw])

Search strategy CENTRAL (Cochrane Central Register of Controlled Trials)

#1 (pancreas OR pancreatic OR pancreato*) NEAR (resection* OR removal OR surger* OR surgical OR laparotom* OR enucleation* OR operation* OR operated) #2 MeSH descriptor: [Pancreas] explode all trees and with qualifier(s): [surgery - SU] #3 (pancreaticoduodenectom* OR pancreatoduodenectom* OR pancreatoÔÇÉduodenectom* OR duodenopancreatectom* OR pancreatectom* OR Whipple OR Kausch-Whipple OR ppWhipple OR dpphr OR PPPD) #4 MeSH descriptor: [Pancreaticoduodenectomy] explode all trees #5 MeSH descriptor: [Pancreatic Diseases] explode all trees and with qualifier(s): [surgery - SU] #6 MeSH descriptor: [Pancreatectomy] explode all trees #7 #1 OR #2 OR #3 OR #4 OR #5 OR #6

Study or Subgroup	Somatostatine Events	Somatostatine Total	Control Events	Control Total	Weight	Odds ratio M-H, Random, 95% CI
**1.4.1 Pancreatoduodenectomy (PD)**						
Klempa et al	1	12	2	12	1.1%	0.45 [0.04 , 5.81]
Montorsi et al.	8	76	10	67	6.7%	0.67 [0.25 , 1.81]
Beguiristain et al	2	21	5	14	2.1%	0.19 [0.03 , 1.17]
Lowy et al.	16	57	11	53	8.5%	1.49 [0.62 , 3.59]
Briceno-Delgado et al.	0	16	5	18	0.8%	0.07 [0.00 , 1.47]
Yeo et al.	11	104	10	107	8.1%	1.15 [0.47 , 2.83]
Gouillat et al.	4	38	10	37	4.2%	0.32 [0.09 , 1.13]
Shan et al.	2	27	2	27	1.7%	1.00 [0.13 , 7.67]
Suc et al.	18	92	18	85	12.0%	0.91 [0.44 , 1.88]
Kurumboor et al.	6	55	10	54	5.6%	0.54 [0.18 , 1.60]
Hesse et al.	5	41	3	39	3.0%	1.67 [0.37 , 7.50]
Kollmar et al.	3	35	1	32	1.3%	2.91 [0.29 , 29.47]
Fernandez-Cruz et al.	2	32	3	30	2.0%	0.60 [0.09 , 3.87]
Wang et al.	11	30	14	30	6.3%	0.66 [0.24 , 1.86]
Belayev et al.	2	10	2	9	1.4%	0.88 [0.10 , 7.95]
Allen et al.	11	111	23	109	10.8%	0.41 [0.19 , 0.89]
El Nakeeb et al.	4	52	4	52	3.3%	1.00 [0.24 , 4.23]
You et al.	1	29	5	30	1.4%	0.18 [0.02 , 1.63]
Cao et al.	13	99	25	100	11.8%	0.45 [0.22 , 0.95]
**Subtotal (95% CI)**		**937**		**905**	**92.0%**	**0.67 [0.51 , 0.88]**
Total events:	120		163			
Heterogeneity: Tau² = 0.00; Chi² = 18.16, df = 18 (P = 0.45); I² = 1%						
Test for overall effect: Z = 2.86 (P = 0.004)						
**1.4.2 Distal Pancreatectomy (DP)**						
Montorsi et al.	2	33	7	33	2.5%	0.24 [0.05 , 1.25]
Suc et al.	3	30	2	23	2.0%	1.17 [0.18 , 7.63]
Allen et al.	3	41	9	39	3.5%	0.26 [0.07 , 1.06]
**Subtotal (95% CI)**		**104**		**95**	**8.0%**	**0.37 [0.15 , 0.93]**
Total events:	8		18			
Heterogeneity: Tau² = 0.00; Chi² = 1.93, df = 2 (P = 0.38); I² = 0%						
Test for overall effect: Z = 2.12 (P = 0.03)						
**Total (95% CI)**		**1041**		**1000**	**100.0%**	**0.64 [0.49 , 0.84]**
Total events:	128		181			
Heterogeneity: Tau² = 0.01; Chi² = 21.63, df = 21 (P = 0.42); I² = 3%						
Test for overall effect: Z = 3.28 (P = 0.001)						
Test for subgroup differences: Chi² = 1.52, df = 1 (P = 0.22), I² = 34.4%						

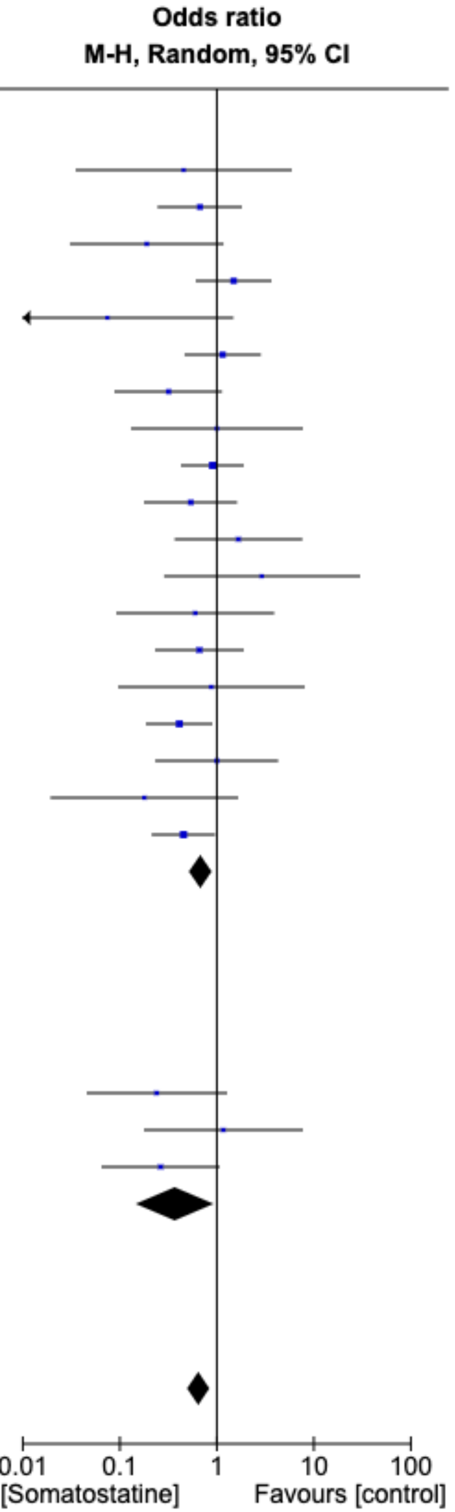

Definitions of primary outcome, POPF and DGE

Somatostatine

Trial	Definition	primary outcome
**Cao 2021**	ISGPS POPF; pancreatectomy-releated complications: ISGPS; adverse events graded according CTCAE version 5.0	CR-POPF (Grade B and C)
**Travainen 2020**	ISGPS : POPF, DGE, PPH, postpancreatectomy complications; CCI (Slankamenac 2014)	Comprehensive Complication Index (CCI)
**You 2019**	ISGPS POPF;	Exocrine secretion and POPF
**El Nakeeb 2018**	ISGPS POPF, DGE ; Bile leak: International Study group of Liver surgery (ISGLS) 2011; Postoperative morbidity: Clavien-Dindo-Classification	rate of POPF
**Kurumboor 2015**	POPF: ISGPS	POPF, complications and death
**Allen 2014**	MSKCC Surgical Secondary Events system: POPF, pancreatic anastomic leak, intraabdominal abscess ISGPS: POPF	grade 3 or higher pancreatic fistula, leak, or abscess at 60 days postoperatively, as defined by the MSKCC Surgical Secondary Events system
**Belyaev 2013**	ISGPS: POPF, DGE, PPH	increased pancreatic hardness
**Fernández-Cruz 2013**	POPF: ISGPS	pancreatic remnant exocrine secretion and the rate of POPF
**Wang 2013**	POPF: ISGPS	POPF and other postoperative complications
**Katsourakis 2010**	POPF: ISGPS	ultra-structure of exocrine pancreatic cells
**Kollmar 2008**	POPF: ISGPS; DGE Lermite et. al 2007	POPF, DGE
**Closset 2008**	nr	
**Ramos-De la Medina 2006**	nr	pancreatic exocrine secretions
**Hesse 2005**	**POPF**: by drainage of more than 100 ml/day of amylase-rich fluid, which had to be more than 5 times the upper limit of normal serum amylase after day 3 and persisting beyond postoperative day 7 with rising temperature and preseptic conditions.	POPF and other postoperative complications

**Suc 2004**	**POPF**: either chemically as fluid obtained through drains or percutaneous aspiration containing at least 4 times normal serum values of amylase for 3 days, irrespective of the amount of output and the date of appearance, or clinically and radiologically as anastomotic leaks demonstrated by fistulography or upper gastrointestinal tract hydrosoluble contrast studies in the case of pancreatogastrostomy **PPH**: blood exiting through the nasogastric tube and confirmed on fibroendoscopy or arteriography and intra-abdominal hemorrhage by exteriorization of blood through drains on arteriography or bot	postoperative intra-abdominal complications
**Sarr 2003**	**POPF**: Drainage fluid (beginning on or after POD 5) of both > 30 mL/ day and amylase or lipase activity > 5x upper limits of the normal serum value **Perianastomic fluid collection**: sterile fluid collection requiring drainage operative or percutaneous) with an amylase or lipase activity > 5x upper limits of normal serum value. **perianastomic abcess:** Pus with an amylase or lipase activity ≥ upper limits of the normal serum value	pancreatic-related complication
**Shan 2003**	**POPF:** amylase-rich fluid with drain fluid volume greater than 10 mL/day, persistent elevation of the drain amylase level and three times higher than the serum level for longer than 7 days **Postoperative pancreatitis**: an elevation of serum amylase from POD 4, with morphologic evidence by CT, or laparotomy; **Leakage of pancreaticojejunostomy**: confirmed by radiologic contrast method or repeat laparotomy; **Perianastomotic fluid collection:** intraperitoneal sterile fluid with or without amylase elevation; **Intraabdominal abscess:** infected fluid collection confirmed by positive bacterial culture, with or without amylase fluid collection, detected by CT-guided aspiration or re peat laparotomy; **Systemic complications**: shock, sepsis, respiratory failure, renal failure, or bleeding, was recorded according to its relationship to the remnant pancreas	pancreatic stump-related complications
**Falconi 2002**	nr	exocrine pancreatic secretion.

**Gouillat 2001**	**POPF**: drain (> 100ml/day) of amylase-rich drainage fluid (> 5x upper the limit of normal serum amylase) after 3 days, persiting after day 12 or is assication with raised temperature (>38°C) or other symptomys requiring further surgery, percutaneous drainage or transfer **biochemical fistula:** asymtomatic fistula, indentified by raised amylase concentration in the drain fluid (>5x upper of normal serum limit) after day 3, for 5 days or more, but which resolved spontaneously before day 12 **intra-abdominal abcess**: infected fluid collection revealed by ultasonography, CT, guided needle aspiration or further surgery. **acute pancreatitis:** increase to at least three times the normal plasma amylase concentration more than 4 days aftert operation, associated with morphological signs using contrast-enhanced CT	exocrine pancreatic secretion.
**Bonora 2001**	nr	postoperative complications
**Yeo 2000**	**POPF**: persistent drainage of 50 mL or more of amylase-rich fluid on or after postoperative day 3 through. The incidence of pancreatic anastomotic leak varies from 5% to 25% in most series.	POPF, complications and death
**Chae 1999**	nr	Exocrine secretion
**Briceno Delgado 1998**	nr	overall complications
**Lowy 1997**	**POPF**: concentration amylase and lipase in the abdominal drain effluent (single aliquot >10 ml/day) that was more than three the serum concentration on or after POD 3, in the absence of clinical or radiographic finding diagnostics of a pancreatic anastomic leak	POPF
**Friess 1995**	nr	overall complications (death, leackage, POPF, Intra-abdominal abscess, intra-abdominal fluid collection (steril), sepsis, bleeding , postoperative acute pancreatitis, renal insufficiency, cardiocirculatory shock)
**Beguiristain 1995**	**POPF**: presence of an amylase concentration higher than 5,000 IU in the abdominal drainage fluid and a drainage volume of more than 1Oml/24 hours. The abdominal drainage fluid and a drainage volume of more than 1Oml./24 hours.	POPF, overall complications

**Monrorsin 1996**	**POPF**: amylase-rich fluid (amylase more than three times normal serum concentration) collected from the peripancreatic abdominal drainage since the postoperative day 3, with a drainage volume of greater than 10 ml/day. **Leakage of pancreatic, biliary or enteric anastomosis:** confirmed by relaparotomy or radiologic contrast methods. **Postoperative acute pancreatitis:** 3x elevation of serum amylase since the postoperative day 4, with morphologic confirmation by CT, ultrasonography, or relaparotomy. **Peripancreatic fluid collection:**intraperitoneal sac containing sterile fluid, with or without amylase **Intraabdominal abscess:** infected fluid collection (with bacteriologic confirmation) with or without the presence of pus and with normal amy- lase fluid concentration, revealed by CT-ultrasono- gram-guided aspiration or laparotomy	
**Pederzoli 1994**	**POPF**: Drain output of fluid with amylase content more than three times the maximum normal value exceeding 10 ml per 24 h for at least 4 days from day 4 after operation **Abdominal abscess**: Collection of pus or infected fluid confirmed by either ultrasound- or CT-guided aspiration and culture, or by a second laparotomy **abdoininal fluid collection**: Collection of fluid at least 5 cm in diameter, diagnosed by ultrasonography or CT and not identifiable as an abscess **Acute pancreatitis**: Increase to at least three times normal of plasma amylase or lipase level more than 4 days after operation and confirmed by ultrasonography or CT **Sepsis:** Presence of at least four of the following positive blood culture, rectal temperature >38.5"C, leucocytosis > 12000 white blood cells/mm3 or leucopenia <4OOO white blood cells/mm3. thrombocytopenia < 15OOOO platelets/mm3, metabolic acidosis (base excess > -4) **Bleeding:** Need for replacement with at least 4 units of packed cells (lo00 ml) at least 24 h after the end of operation	
**Tulassay 1993**	nr	postoperative increase of pancreatic enzyme

**Buechler 1992**	**leakage of pancreatic, biliary, or intestinal anastomosis:** as determined by radiograph or intraoperative finings POPF: concentration of amylase and lipase in the drain fluid more than POD 3 of more than 3x times the serum concentration and the drain volume more than 10ml/24h at thesame time **intra-abdominal abscess**: pus collection or an infected fluid collection (steril) revealed by ultrasound or CT guided needle aspiration and microbiological culture or demostrated by relaparotomy. **intra-abdominal fluid collection:** collection of 5x5 cm in diameter in ultrasound or CT that did not qualify as an abscess	
**Lange 1992**	nr	postoperative pancreatic drain fluid; POPF
**Buccoliero 1992**	nr	complications
**Klempa 1991**	nr	exocrine pancreatic secretion
**Corticosteroids**		
**Trial**	**Primary outcome**	**Definition**
**Tarvainen 2020**	Comprehensive Complication Index (CCI)	ISGPS : POPF, DGE, PPH, postpancreatectomy complications; CCI (Slankamenac 2014)
**Antila 2019**	Overall complications, POPF	**POPF**: ISGPS; **Overall complications**: Clavien-Dindo; **Mortality**: 90-days after surgery;
**Laaninen 2016**	post-pancreaticoduodenectomy complications	**POPF**: ISGPS; **DGE**: ISGPS; **PPH**: ISGPS; C**omplications**: Clavien-Dindo-Classification
**PERT**		
**Trial**	**Primary outcome**	**Definition**
**Kim 2020**	body weight, nutritional status, and quality of life	QoL
**Yasukawa 2020**	incidence of NAFLD within 1 year	**Non-alcoholic-fatty-liver-disease**: defined as a liver-to-spleen attenuation ratio ≤ 0.9
**Satoi 2026**	frequency of NAFLD development	**Non-alcoholic-fatty-liver-disease:** liver-to-spleen attenuation ratio less than 0.9 on CT within 12 months after starting treatment
**Seiler 2013**	CFA from baseline to end of double-blind treatment	Pancreatic exocrine insufficiency (PEI): baseline coefficient of fat absorption (CFA) <80%
**Farkas 2001**	exocrine function	nr
**Neoptolemos 1999**	stool fat excretion, stool volume, and clinical symptoms	nr
**Van Hoozen 1997**	efficacy of pancreatic enzyme supplementation in maintaining postoperative digestion and nutrition	nr
**Erythromycin**		
**Trial**	**Primary outcome**	**Definition**

**Ohwada 2001**	DGE	DGE: a nasogastric tube left in place for 10 or more days, emesis after nasogastric tube removal, reinsertion of nasogastric tube, postoperative use of prokinetic agents after postoperative day 10, or failure to progress with diet
**Yeo 1993**	DGE	DGE: a nasogastric tube left in place for 10 or more days, emesis after nasogastric tube removal, reinsertion of nasogastric tube, postoperative use of prokinetic agents after postoperative day 10, or failure to progress with diet
**PPI**		
**Trial**	**Primary outcome**	**Definition**
**Jang 2013**	Prevention of Pancreatic Atrophy	nr
**Toyota 1999**	gastric stasis	nr
**Glucose Control**		
**Trial**	**Primary outcome**	**Definition**
**van Veldhuisen 2021**	Median percentage of time spent in euglycemia	blood glucose levels between 70 and 180 mg/dL (3.9-10.0 mmol/L) during a 7-day treatment with the BIHAP in patients after a total pancreatectomy compared with current diabetes care
**Okayabashi 2009**	Incidence of severe hypoglycemia	Hypoglycemia: <40 mg/dL during the intensive care period following pancreatic resection in patients monitored with the artificial pancreas

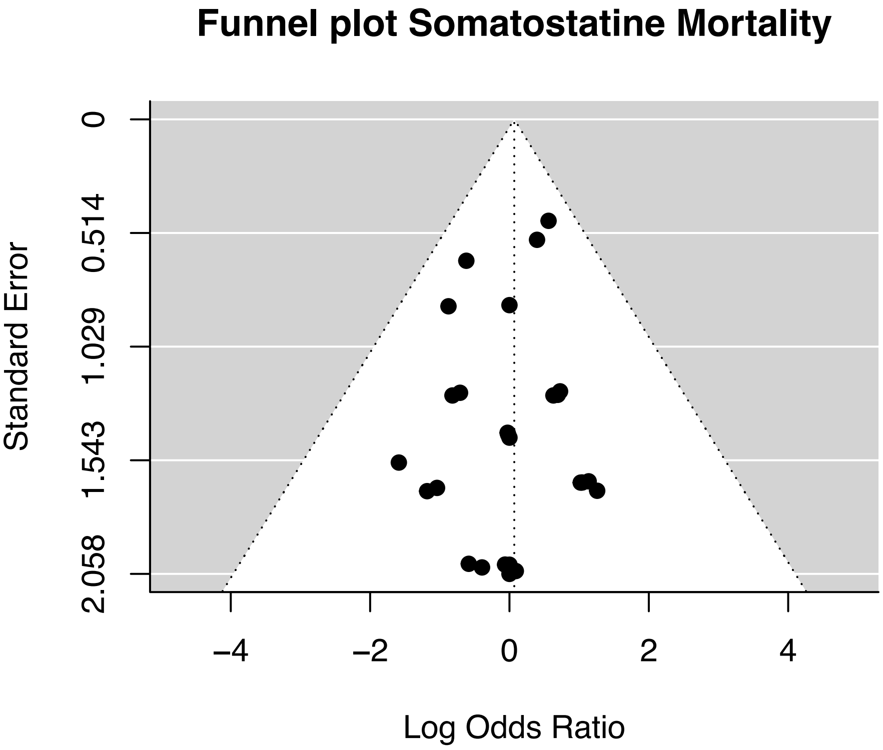

Funnel plot Somatostatine Mortality
Standard Error
0
0.514
1.029
1.543
2.058
−4
−2
0
2
4
Log Odds Ratio

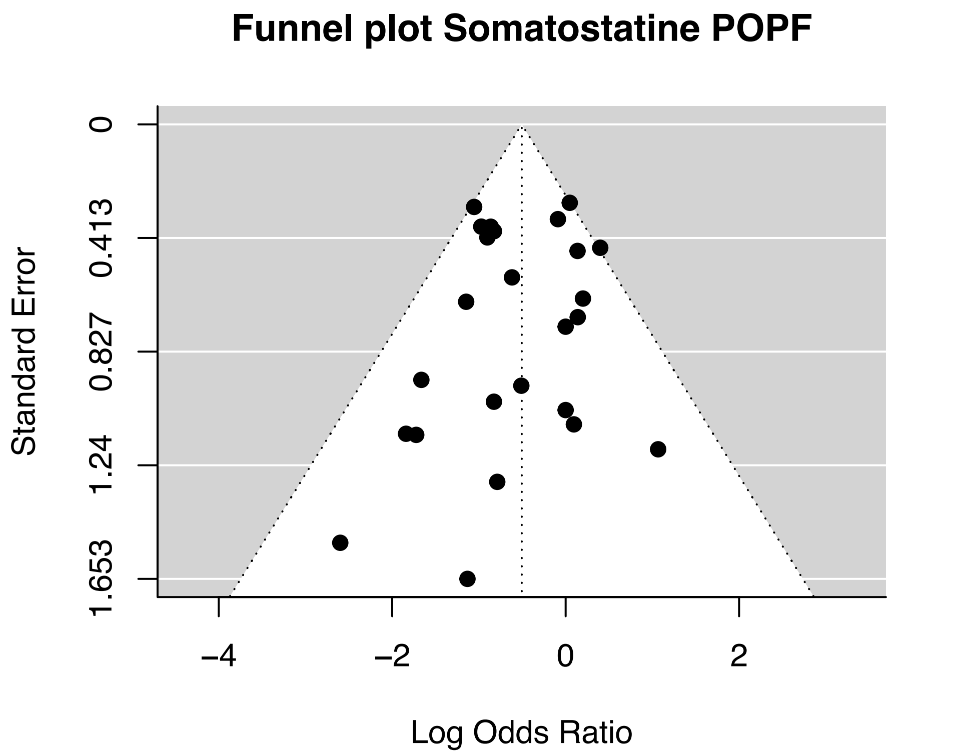

Funnel plot Somatostatine POPF
Standard Error
0
0.413
0.827
1.24
1.653
−4
−2
0
2
Log Odds Ratio

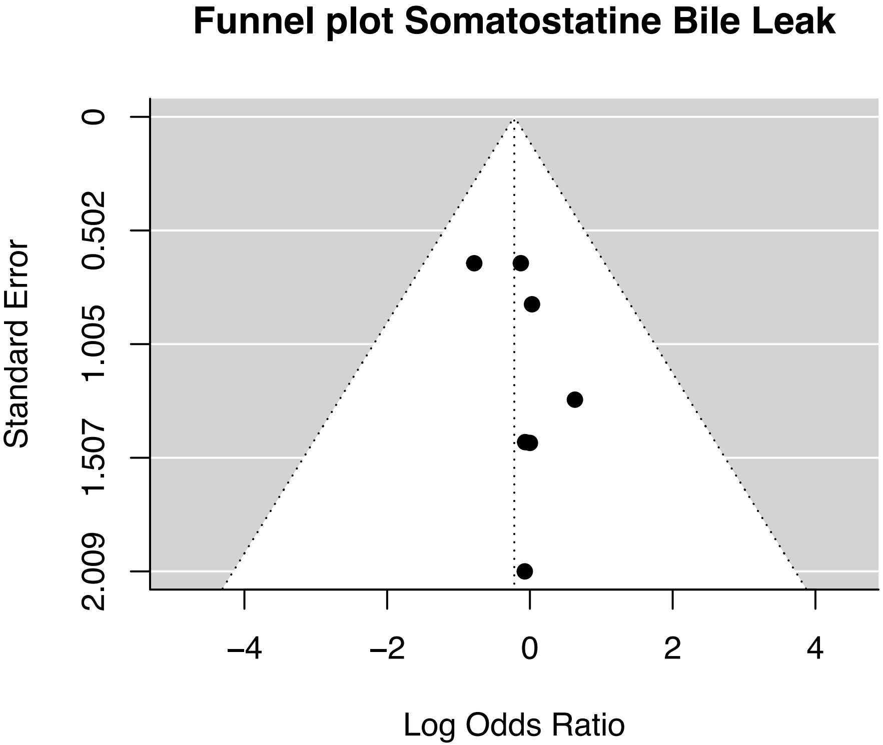

Funnel plot Somatostatine Bile Leak
Standard Error
0
0.502
1.005
1.507
2.009
−4
−2
0
2
4
Log Odds Ratio

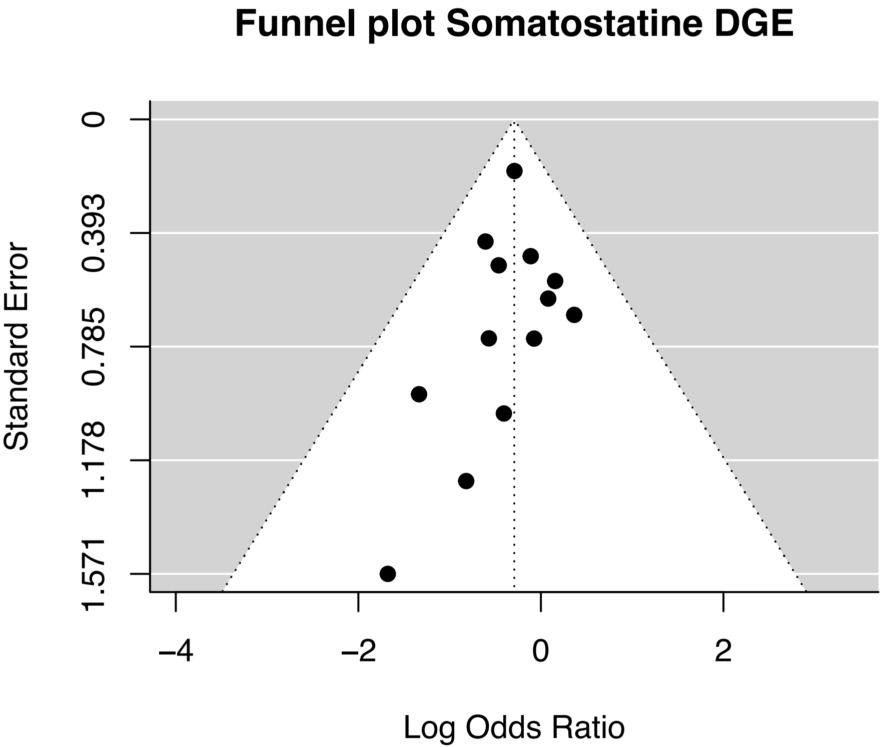

Funnel plot Somatostatine DGE
Standard Error
0
0.393
0.785
1.178
1.571
−4
−2
0
2
Log Odds Ratio

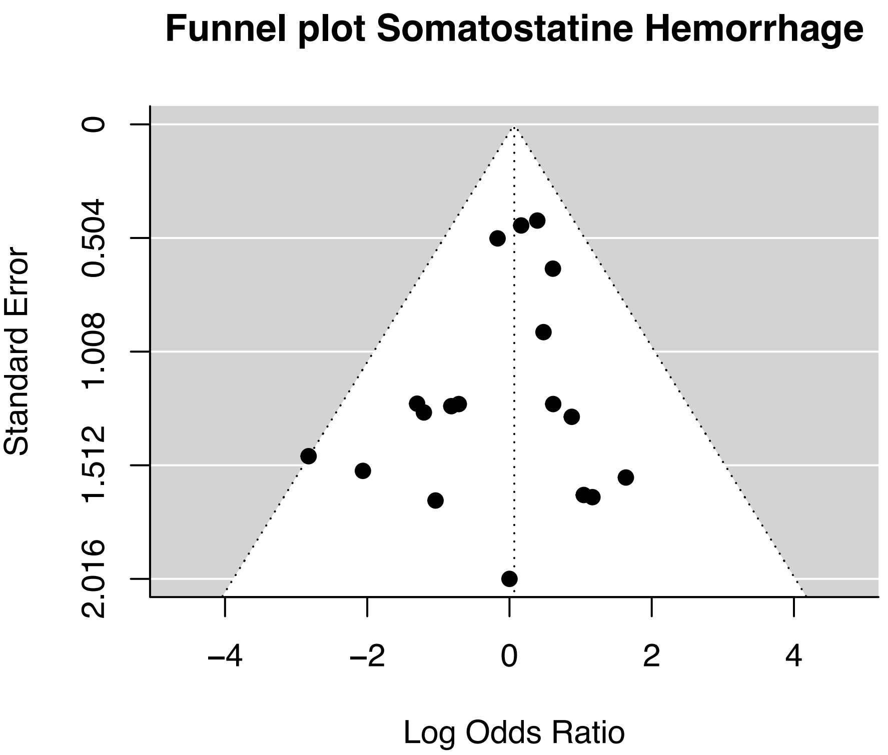

Funnel plot Somatostatine Hemorrhage
Standard Error
0
0.504
1.008
1.512
2.016
−4
−2
0
2
4
Log Odds Ratio

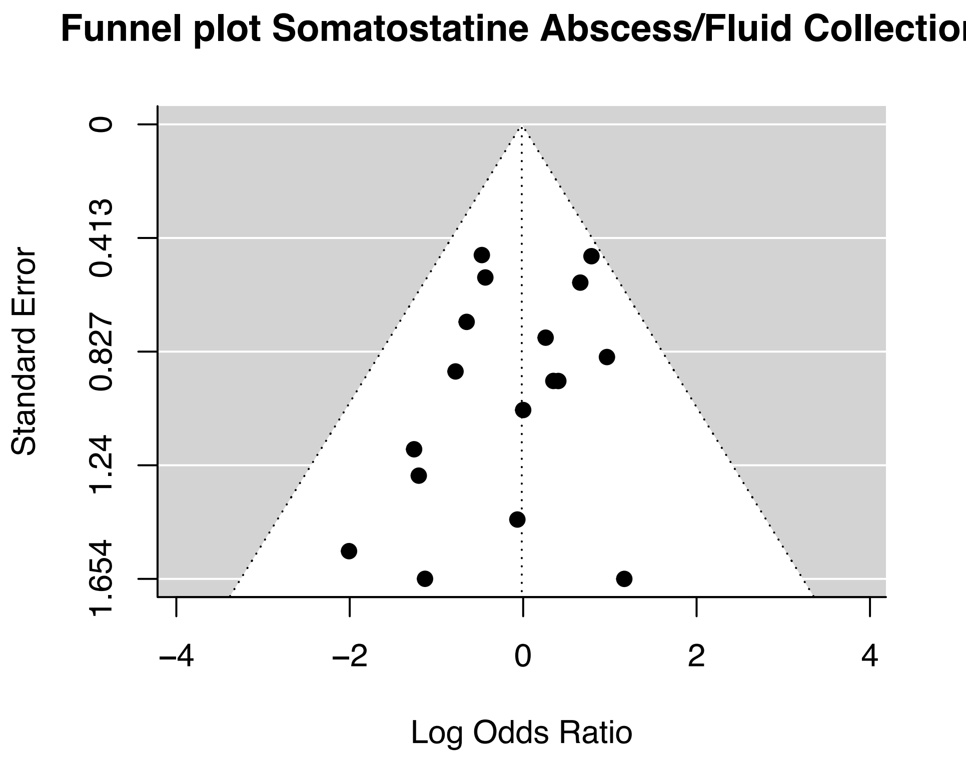

Funnel plot Somatostatine Abscess/Fluid Collection
Standard Error
0
0.413
0.827
1.24
1.654
−4
−2
0
2
4
Log Odds Ratio

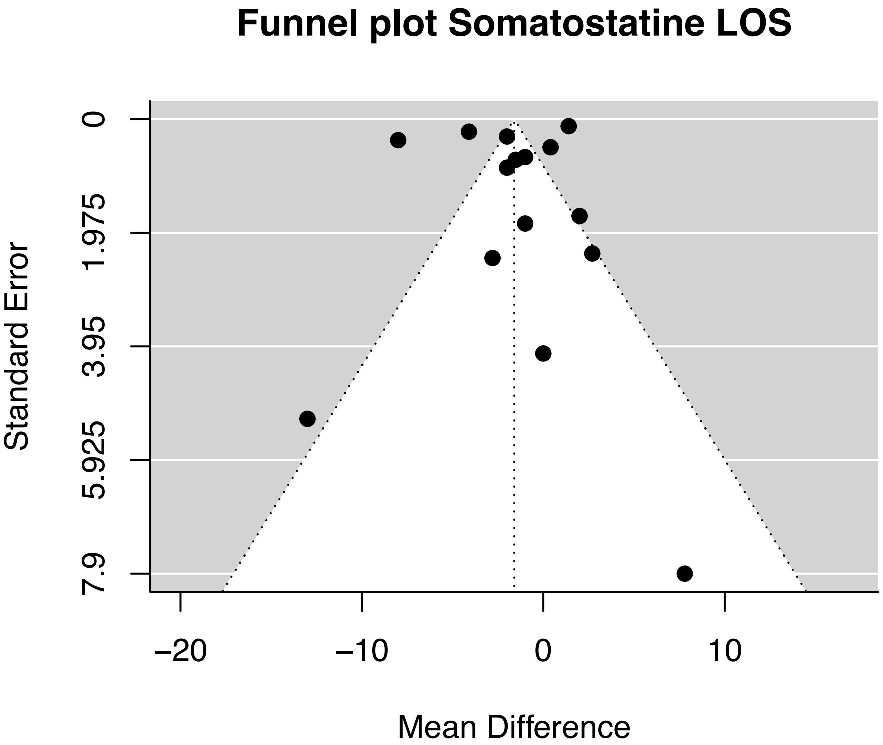

Funnel plot Somatostatine LOS
Standard Error
0
1.975
3.95
5.925
7.9
−20
−10
0
10
Mean Difference

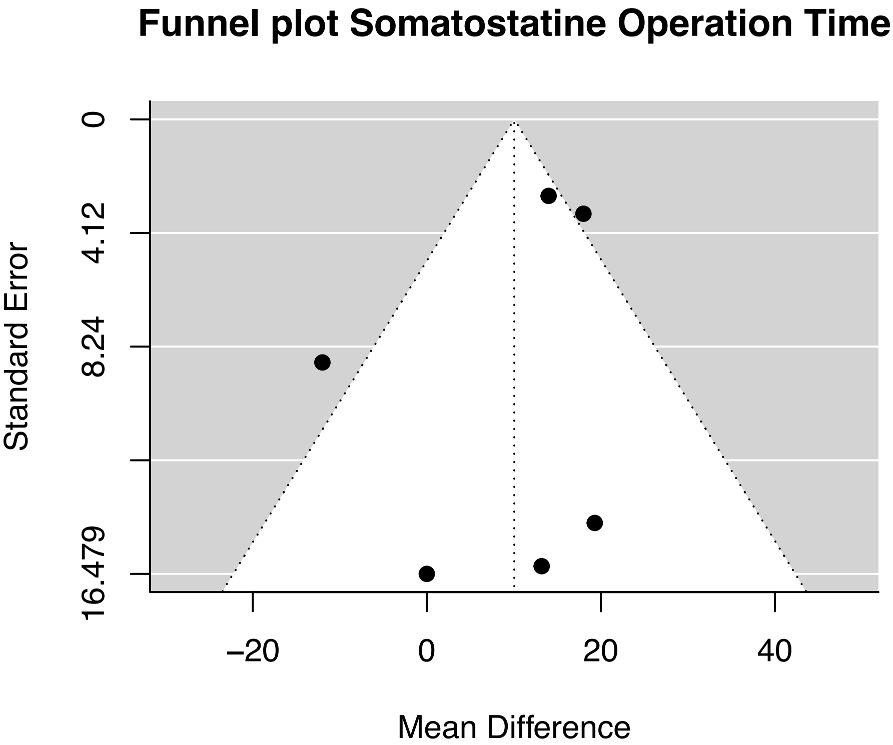
Funnel plot Somatostatine Operation Time
Standard Error
0
4.12
8.24
16.479
−20
0
20
40
Mean Difference

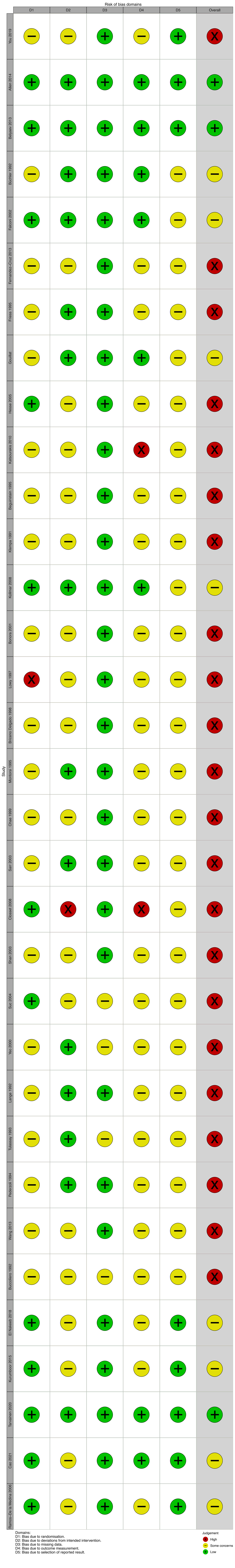
Risk of bias domains
D1
D2
D3
D4
D5
Overall
Study
Yau 2019
Allen 2014
Betjeen 2013
Büchler 1992
Falcon 2002
Fernandez-Cruz 2013
Friess 1995
Gouillat
Hesse 2005
Katsourakis 2010
Beguiristain 1995
Klempa 1991
Kollmar 2008
Bonora 2001
Lowy 1997
Bonerio Delgado 1998
Montorsi 1995
Chen 1999
Sarr 2003
Closset 2008
Shan 2003
Suc 2004
Yeo 2000
Lange 1992
Tulassay 1993
Pederzoli 1994
Wang 2013
Buccoliero 1992
El Nakeeb 2018
Kurumboor 2015
Tarvainen 2020
Cao 2021
Ramos-De la Medina 2006
Domains:
D1: Bias due to randomisation.
D2: Bias due to deviations from intended intervention.
D3: Bias due to missing data.
D4: Bias due to outcome measurement.
D5: Bias due to selection of reported result.
Judgement
High
Some concerns
Low

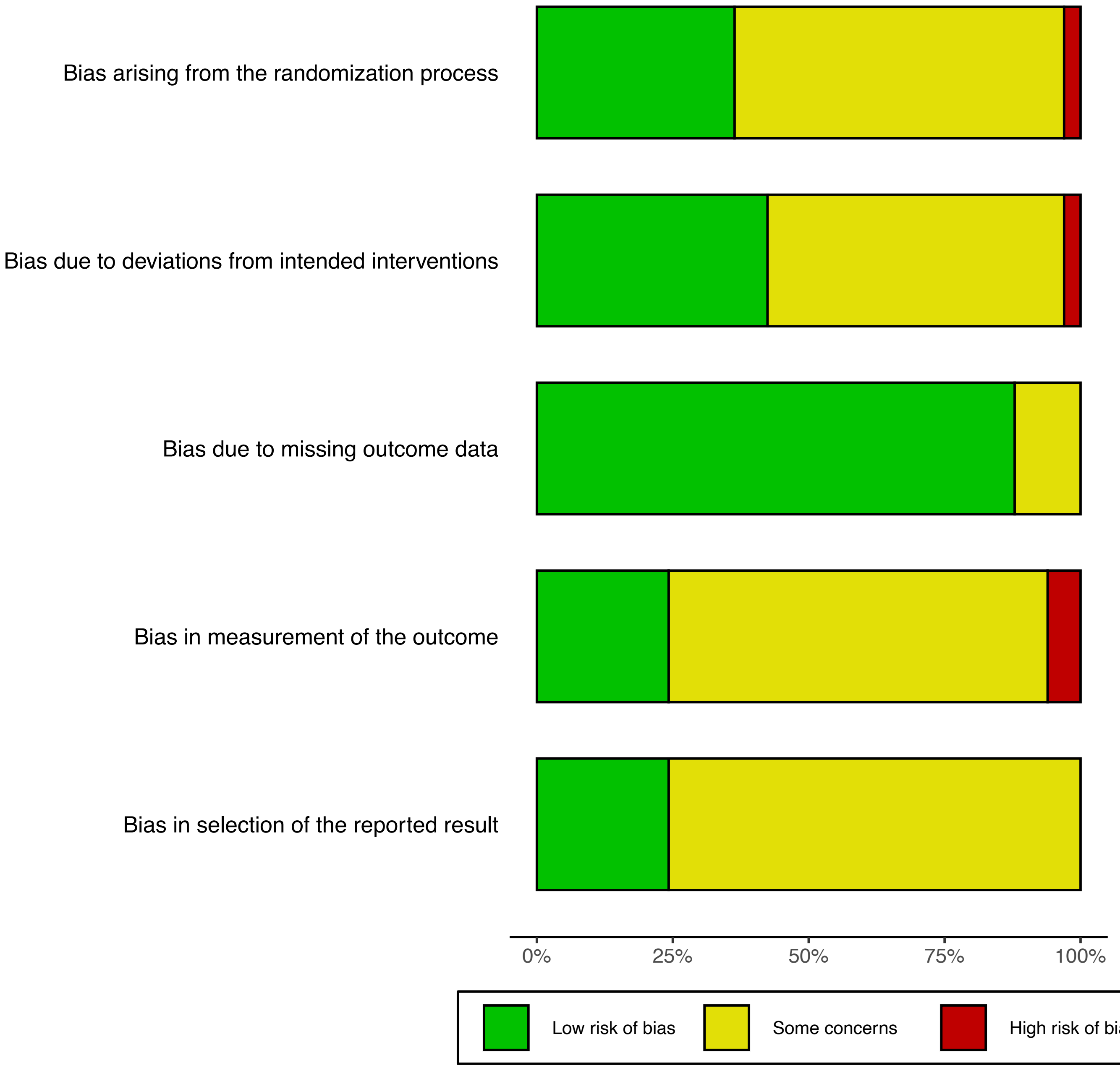
Bias arising from the randomization process
Bias due to deviations from intended interventions
Bias due to missing outcome data
Bias in measurement of the outcome
Bias in selection of the reported result
0%
25%
50%
75%
100%
Low risk of bias
Some concerns

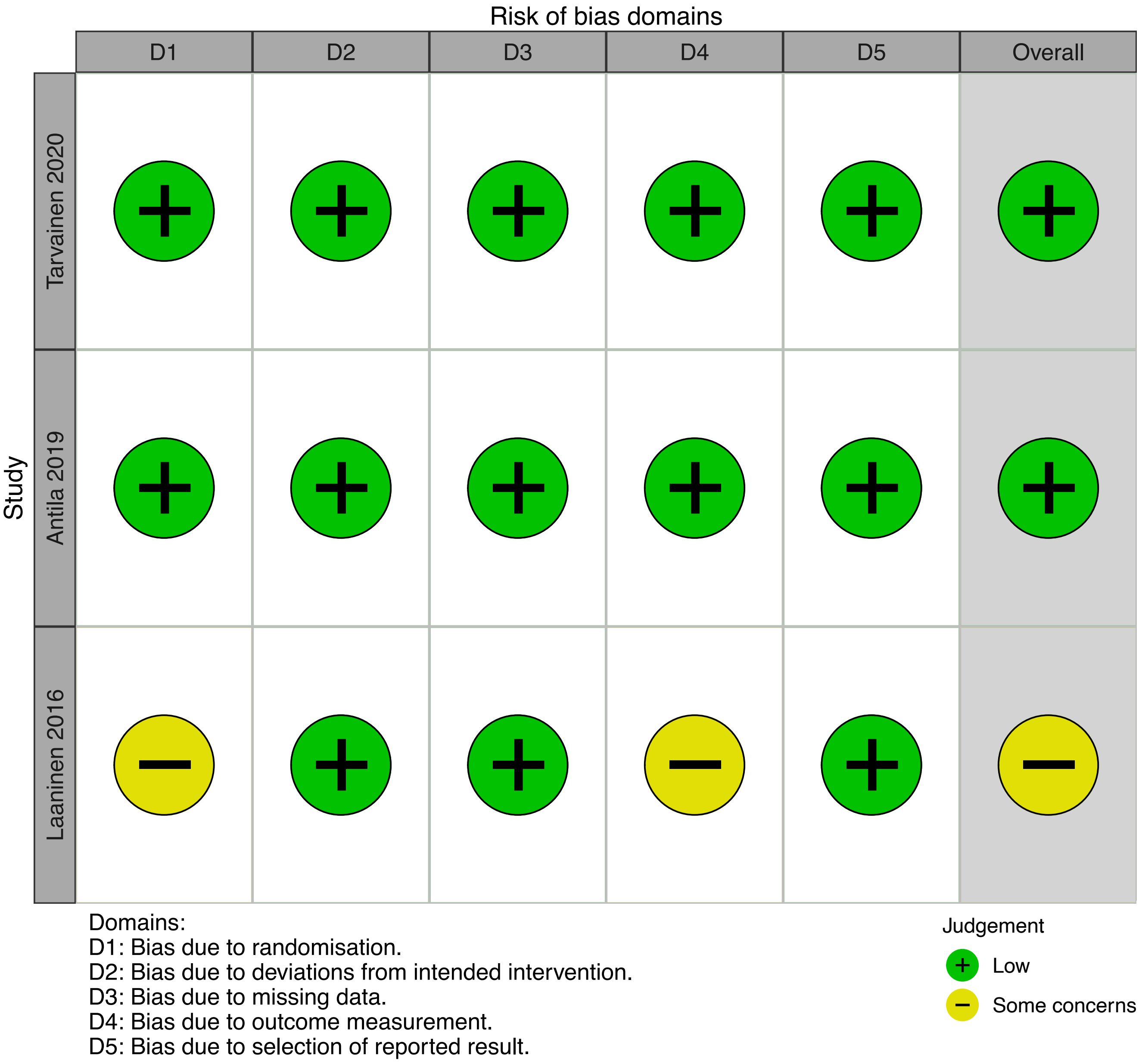
Risk of bias domains
D1
D2
D3
D4
D5
Overall
Study
Tarvainen 2020
Antila 2019
Laaninen 2016
Domains:
D1: Bias due to randomisation.
D2: Bias due to deviations from intended intervention.
D3: Bias due to missing data.
D4: Bias due to outcome measurement.
D5: Bias due to selection of reported result.
Judgement
+ Low
− Some concerns

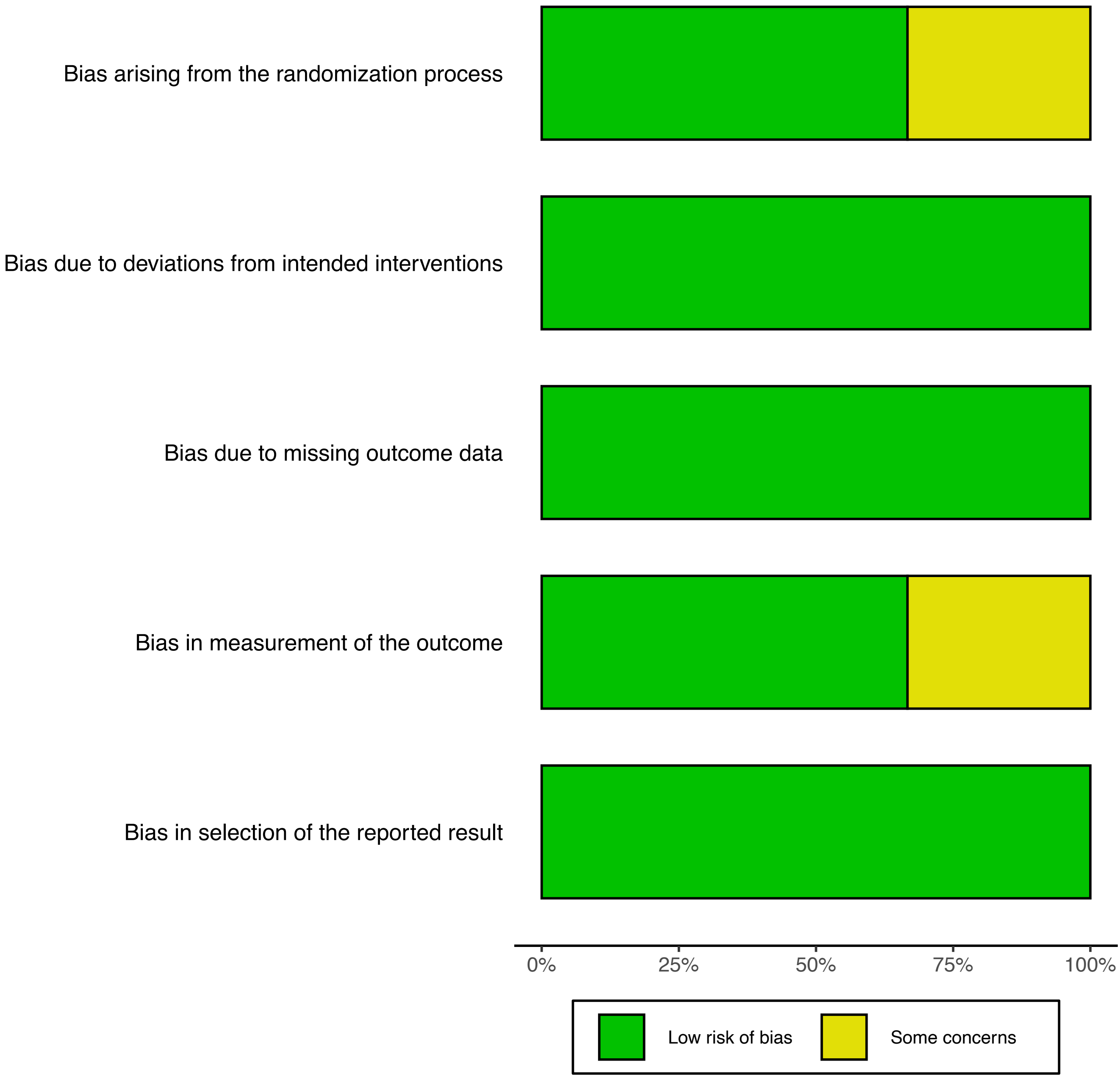

Bias arising from the randomization process
Bias due to deviations from intended interventions
Bias due to missing outcome data
Bias in measurement of the outcome
Bias in selection of the reported result
0%
25%
50%
75%
100%
Low risk of bias
Some concerns

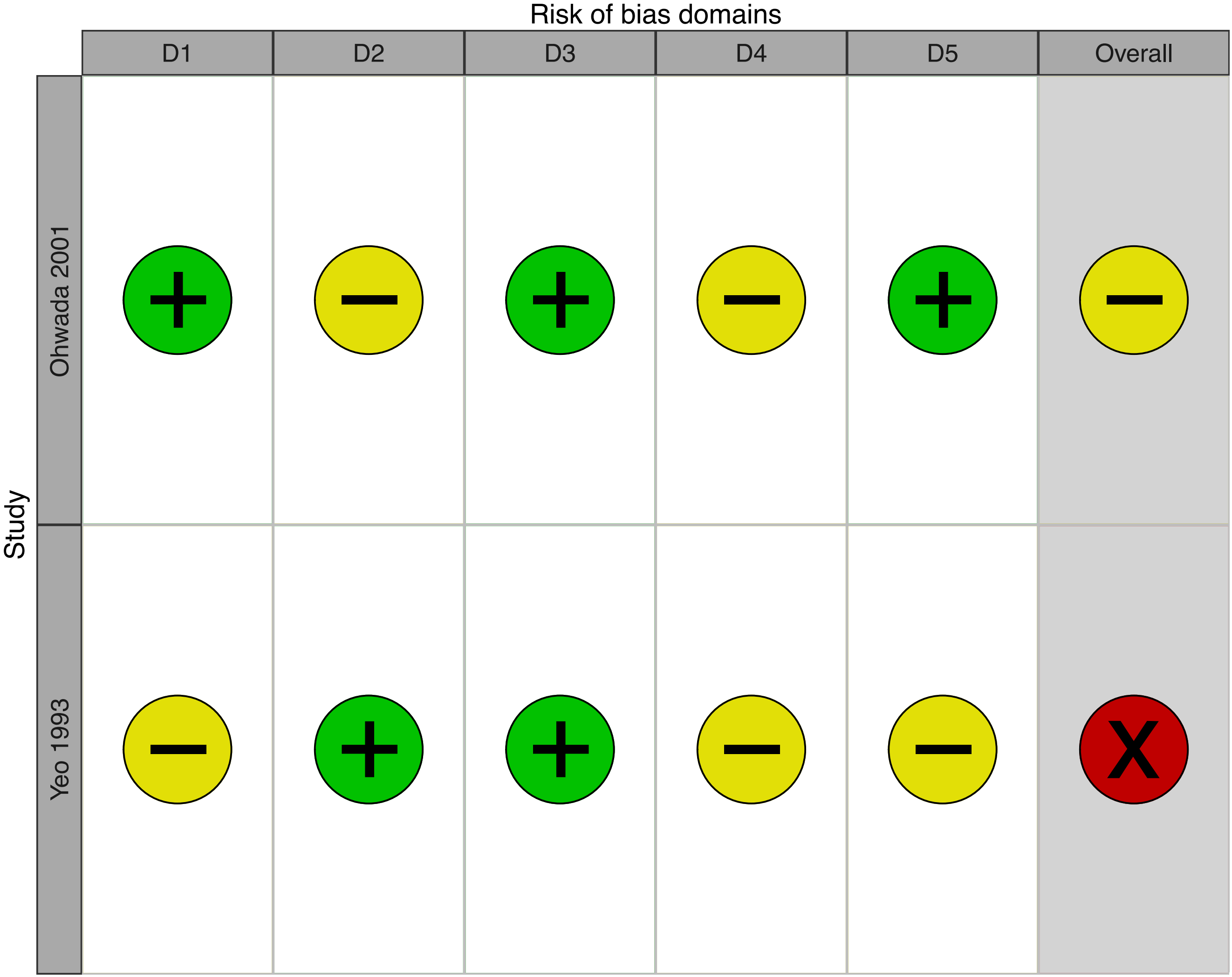

Domains:
D1: Bias due to randomisation.
D2: Bias due to deviations from intended intervention.
D3: Bias due to missing data.
D4: Bias due to outcome measurement.
D5: Bias due to selection of reported result.

Judgement
X High
− Some concerns
+ Low

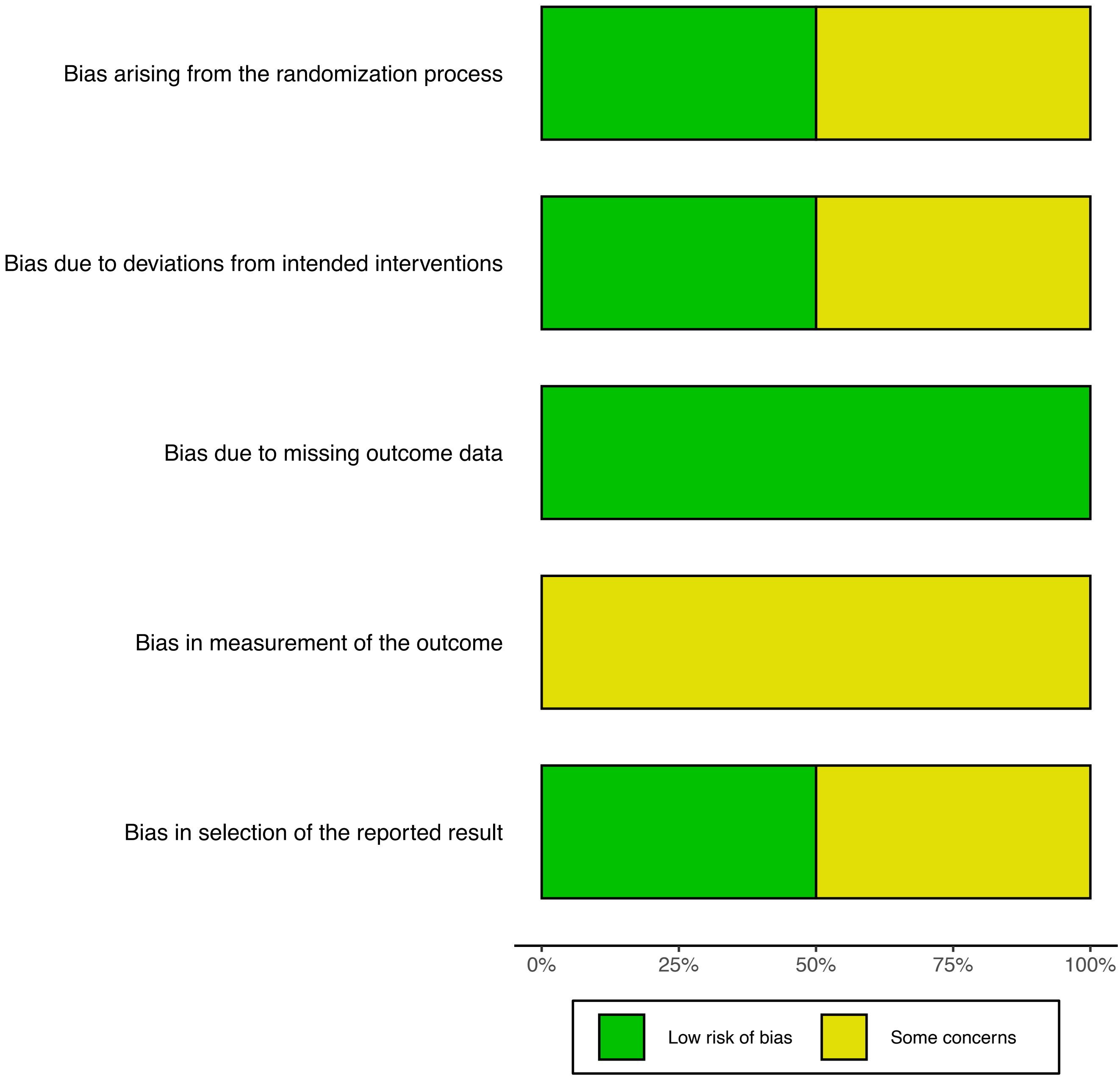
Bias arising from the randomization process
Bias due to deviations from intended interventions
Bias due to missing outcome data
Bias in measurement of the outcome
Bias in selection of the reported result
0%
25%
50%
75%
100%
Low risk of bias
Some concerns

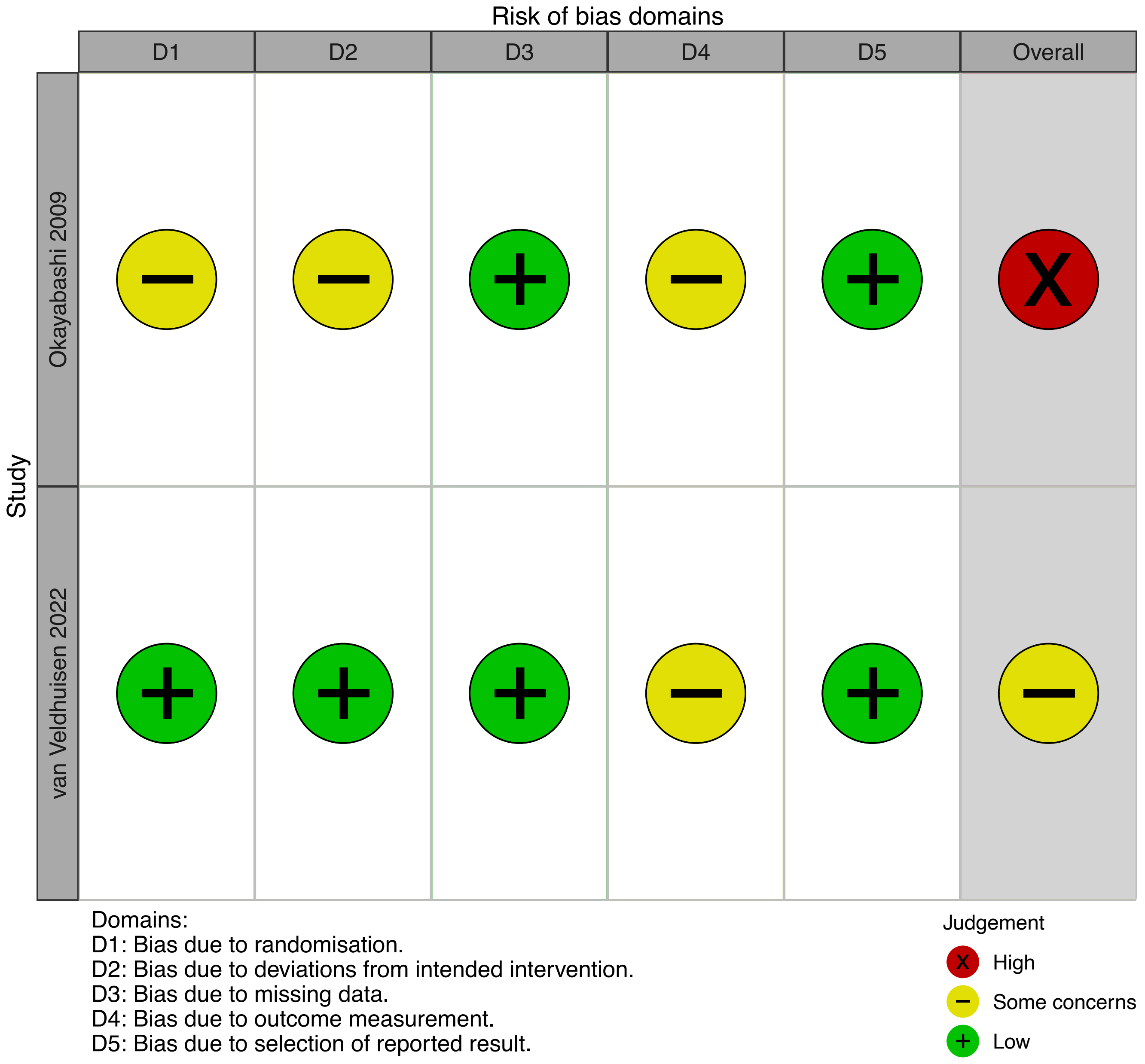
Risk of bias domains
D1
D2
D3
D4
D5
Overall
Study
Okayabashi 2009
van Veldhuisen 2022
Domains:
D1: Bias due to randomisation.
D2: Bias due to deviations from intended intervention.
D3: Bias due to missing data.
D4: Bias due to outcome measurement.
D5: Bias due to selection of reported result.
Judgement
High
Some concerns
Low

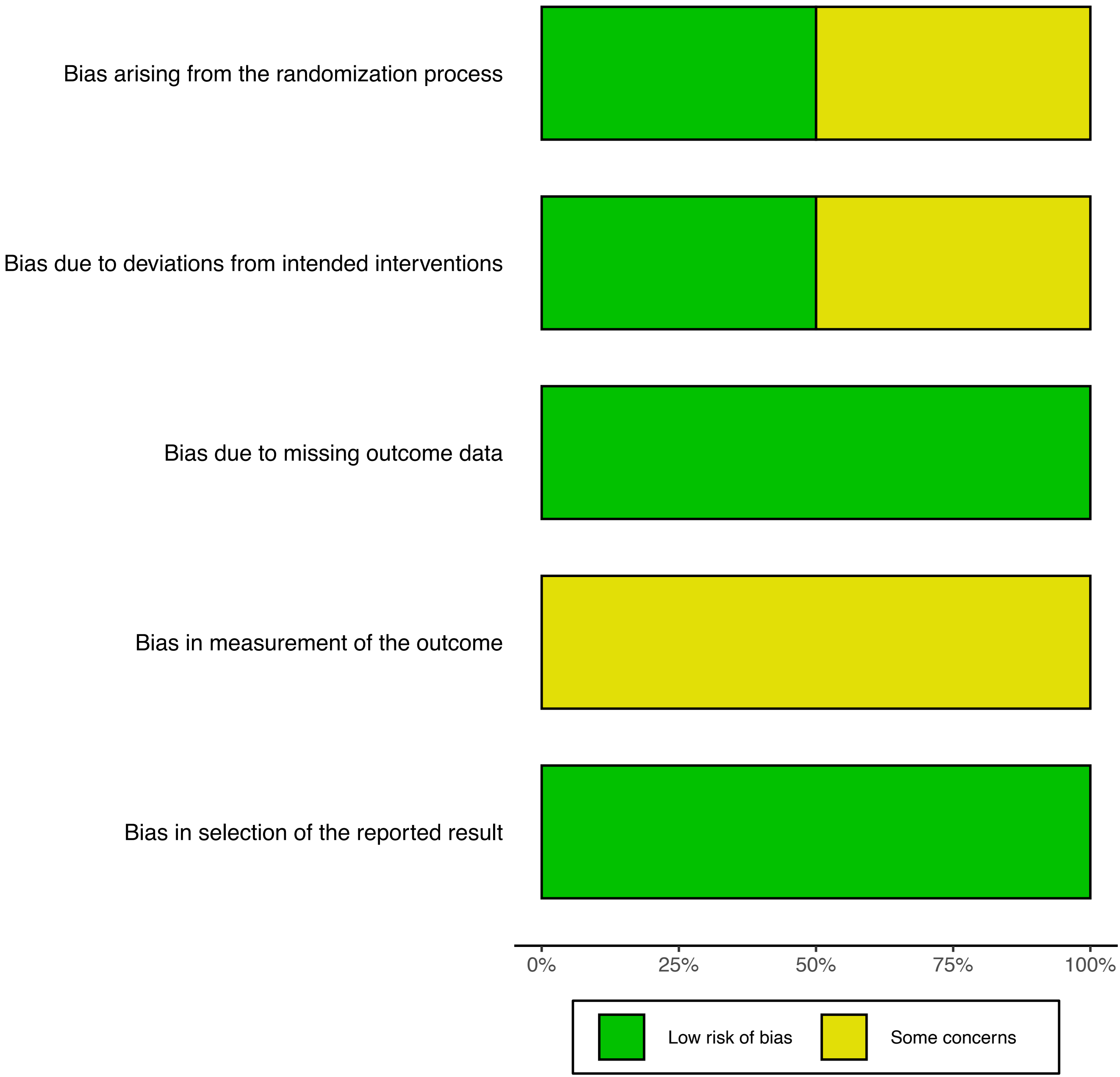

Bias arising from the randomization process
Bias due to deviations from intended interventions
Bias due to missing outcome data
Bias in measurement of the outcome
Bias in selection of the reported result
0%
25%
50%
75%
100%
Low risk of bias
Some concerns

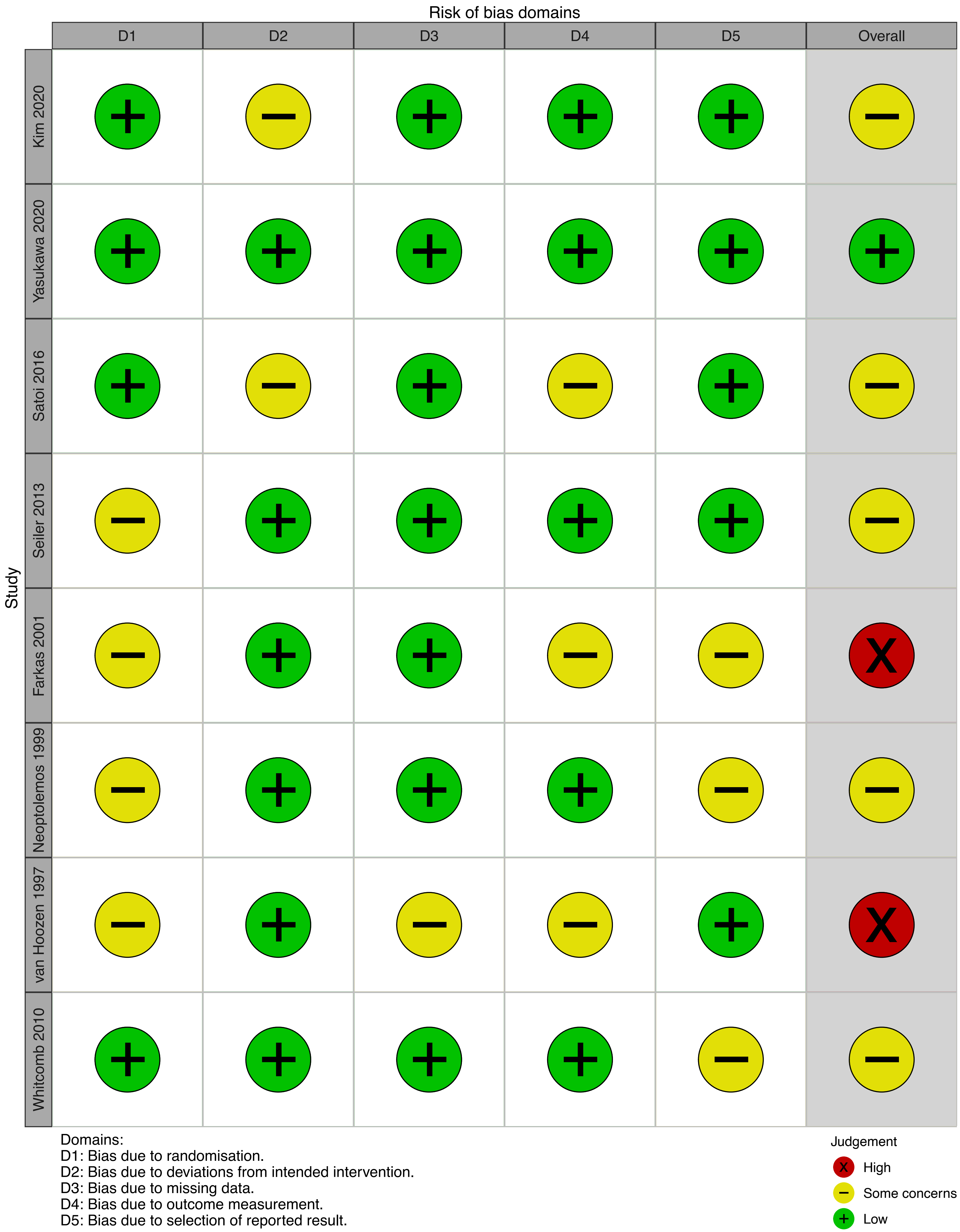
Risk of bias domains
D1
D2
D3
D4
D5
Overall
Study
Kim 2020
Yasukawa 2020
Satoi 2016
Seiler 2013
Farkas 2001
Neoptolemos 1999
van Hoozen 1997
Whitcomb 2010
Domains:
D1: Bias due to randomisation.
D2: Bias due to deviations from intended intervention.
D3: Bias due to missing data.
D4: Bias due to outcome measurement.
D5: Bias due to selection of reported result.
Judgement
High
Some concerns
Low

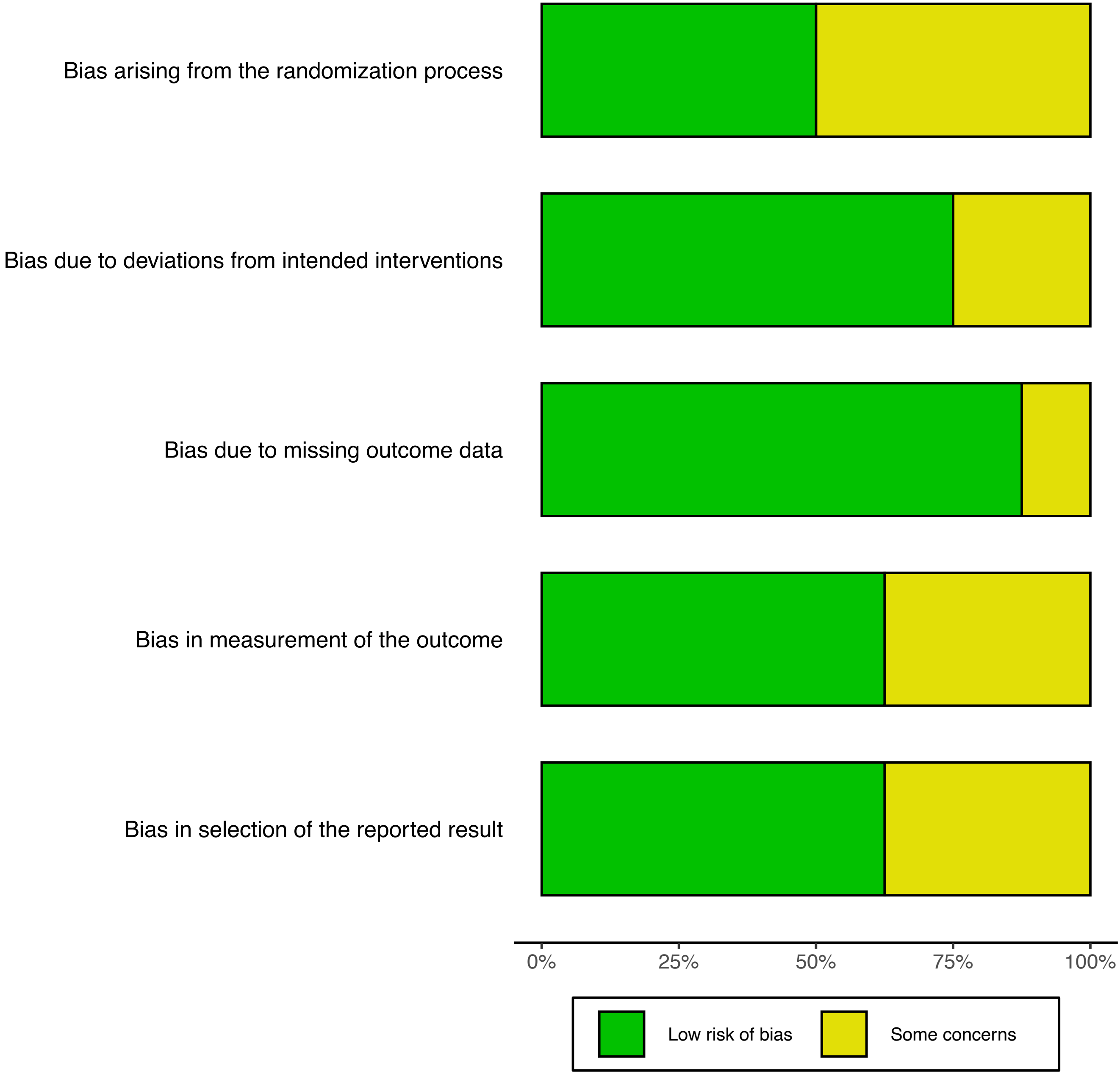
Bias arising from the randomization process
Bias due to deviations from intended interventions
Bias due to missing outcome data
Bias in measurement of the outcome
Bias in selection of the reported result
0%
25%
50%
75%
100%
Low risk of bias
Some concerns

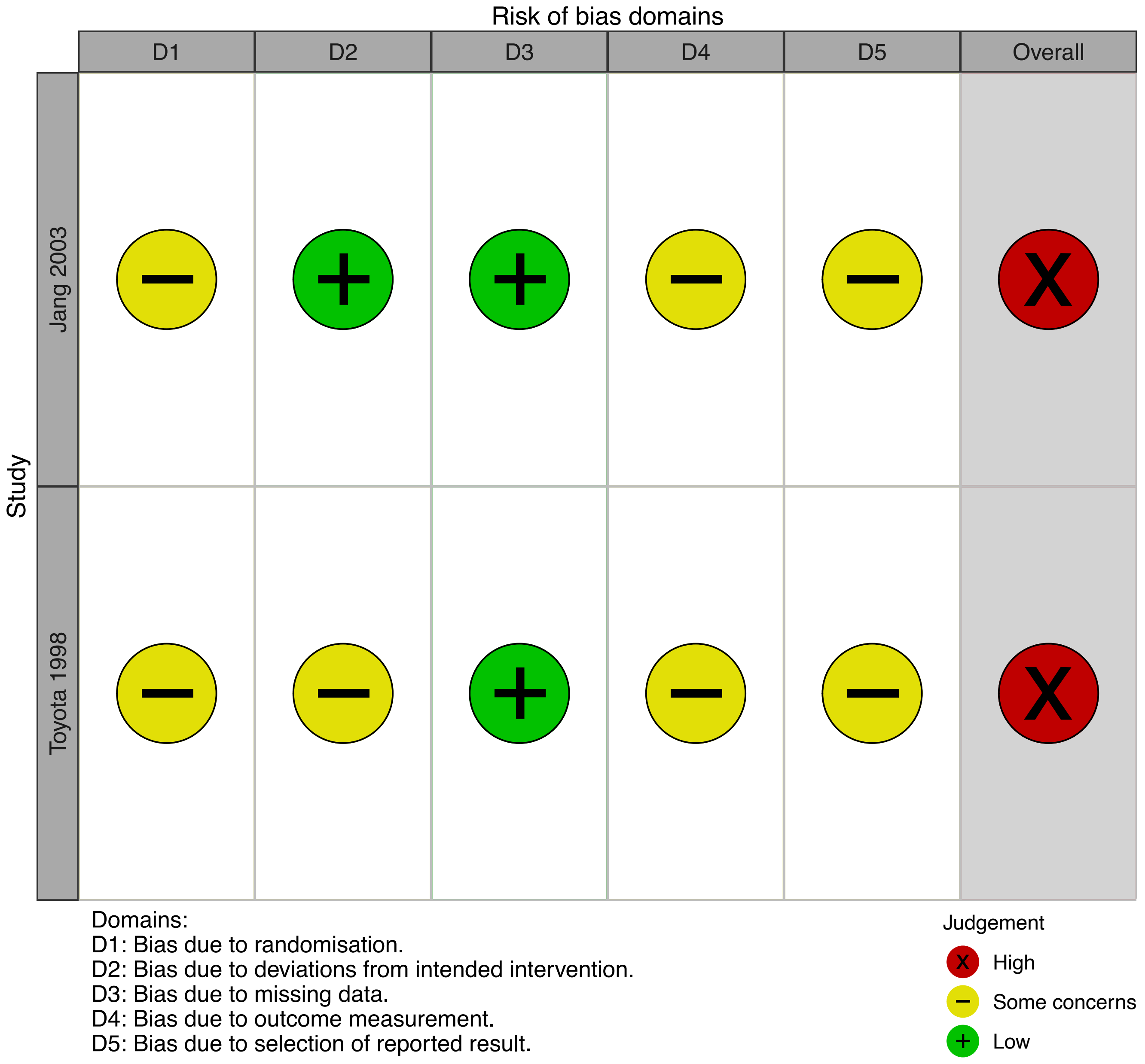
Risk of bias domains
D1
D2
D3
D4
D5
Overall
Study
Jang 2003
Toyota 1998
X
Domains:
D1: Bias due to randomisation.
D2: Bias due to deviations from intended intervention.
D3: Bias due to missing data.
D4: Bias due to outcome measurement.
D5: Bias due to selection of reported result.
Judgement
High
Some concerns
Low

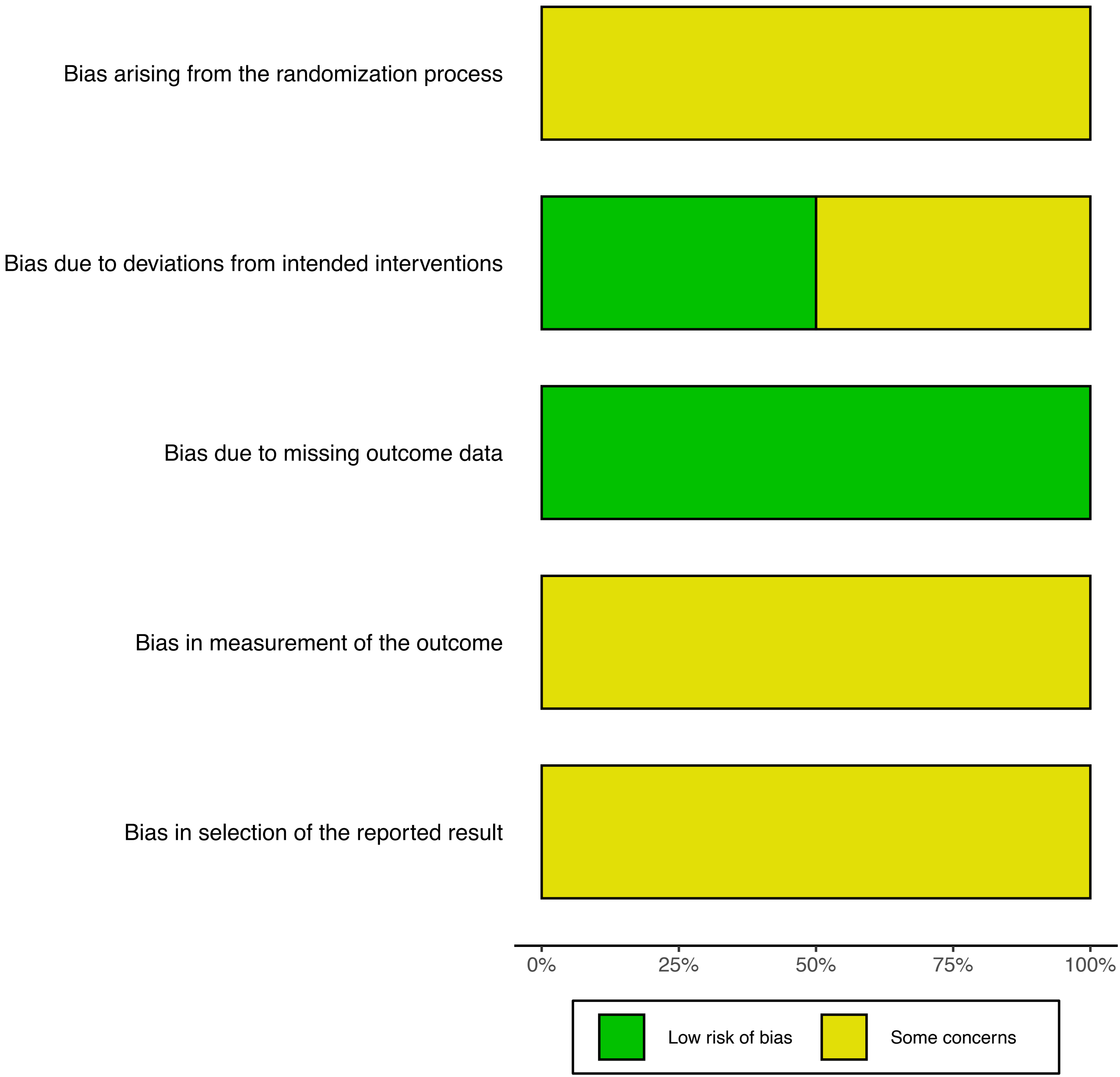

Bias arising from the randomization process
Bias due to deviations from intended interventions
Bias due to missing outcome data
Bias in measurement of the outcome
Bias in selection of the reported result
0%
25%
50%
75%
100%
Low risk of bias
Some concerns
